# Targeting the DNA-repair chromatin-modifier protein SMYD3 as a novel epigenetics-based therapy for gastric cancer

**DOI:** 10.1186/s13046-026-03823-2

**Published:** 2026-09-12

**Authors:** Katia De Marco, Marialaura Latrofa, Giovanna Forte, Martina Lepore Signorile, Elisabetta Di Nicola, Paola Sanese, Candida Fasano, Vittoria Disciglio, Erica Candela, Sergio Coletta, Valentina Grossi, Cristiano Simone

**Affiliations:** 1 Medical Genetics, National Institute of Gastroenterology, IRCCS “Saverio de Bellis” Research Hospital, Castellana Grotte, BA 70013 Italy; 2 Department of Molecular Medicine, Sapienza University of Rome, Rome, 00161 Italy; 3 Core Facility Biobank, National Institute of Gastroenterology, IRCCS “Saverio de Bellis” Research Hospital, Castellana Grotte, BA Italy; 4 Department of Precision and Regenerative Medicine and Jonic Area (DiMePRe- J), University of Bari Aldo Moro, Bari, Italy

**Keywords:** Gastric cancer, SMYD3, DNA damage response, Homologous recombination, PARP inhibitors

## Abstract

**Background:**

SMYD3 is a histone methyltransferase implicated in cancer progression and is overexpressed in several malignancies, including gastric cancer (GC). Building on previous evidence linking SMYD3 to DNA damage repair, we investigated the molecular mechanisms through which SMYD3 regulates double-strand break (DSB) repair in GC and evaluated its therapeutic potential.

**Methods:**

In silico and in vitro analyses were performed to investigate the interactions between SMYD3 and key homologous recombination (HR) proteins, and to evaluate the ATM-mediated phosphorylation of SMYD3. Functional assays assessed the impact of pharmacological SMYD3 inhibition on HR and non-homologous end joining (NHEJ) repair pathways.

**Results:**

Mechanistically, SMYD3 interacted with key HR factors and facilitated chromatin remodeling at damaged sites. ATM phosphorylated SMYD3 at threonine 22, a modification required for HR complex assembly and validated in GC patient samples following neoadjuvant chemotherapy. Inhibition of SMYD3 abolished HR and partially impaired NHEJ. Combined inhibition of SMYD3 and PARP induced synthetic lethality in GC cells, spheroids, and patient-derived organoids, and overcame resistance to olaparib.

**Conclusions:**

These findings reveal the ATM-dependent molecular mechanism by which SMYD3 regulates DNA DSB repair, further supporting SMYD3 as a promising therapeutic target for precision oncology strategies in GC.

**Supplementary Information:**

The online version contains supplementary material available at https://doi.org/10.1186/s13046-026-03823-2.

## Background

Gastric cancer (GC) is a major cause of cancer-related mortality worldwide [1], due to late diagnosis and poor survival outcomes [2]. Currently, chemotherapy is the mainstay of GC treatment, but its efficacy is limited by frequent relapse and the emergence of drug resistance [3]. Although targeted therapies and immune checkpoint inhibitors have improved treatment strategies, options for advanced GC, which is a highly heterogeneous disease, remain limited [2]. Poly ADP-ribose polymerase (PARP) inhibitors (PARPis) are emerging as a promising strategy for GC [4]. Those targeting DNA damage repair pathways exhibit potent antitumor activity in preclinical and clinical studies on GC, especially when ATM or RAD51 expression is low [5]. As a result, olaparib and other PARPis, such as rucaparib, are being investigated in clinical trials for BRCA-mutation-associated GC [6]. Several studies have reported a positive correlation between high SMYD3 levels and cancer progression, suggesting that SMYD3 upregulation is a potential risk factor in the biological behavior and prognosis of GC [7]. Indeed, SMYD3 is highly overexpressed in several types of tumors, including colorectal cancer (CRC), breast cancer, GC, pancreatic cancer, lung cancer, and hepatocellular carcinoma [8]. SMYD3 is overexpressed in GC tissues compared with non-tumor tissues and is positively correlated with STAT3, MMP-9, and TGFß1 expression [9]. SMYD3 was first characterized as a histone H3K4/H4K5 methyltransferase associated with RNA polymerase complexes, which promotes transcriptional activation of genes controlling proliferation, cell cycle progression, and epithelial–mesenchymal transition [10–12]. Beyond transcriptional regulation, SMYD3 also exerts oncogenic functions by methylating non-histone proteins and modulating key signaling pathways involved in cell survival, proliferation, stemness, and self-renewal [13–15]. Emerging evidence suggests a role for SMYD3 in maintaining genomic integrity in cancer cells; it has been reported to participate in the homologous recombination (HR) pathway by modulating the expression of specific HR genes [16]. Consistent with this, our previous study demonstrated that SMYD3 interacts with the HR proteins ATM, CHK2, and BRCA2 and is required for the repair of double-strand breaks (DSBs) [17]. SMYD3 promotes HR complex assembly and facilitates RAD51 recruitment to DNA DSBs in response to endogenous or treatment-induced DNA damage [17]. However, the molecular mechanisms regulating SMYD3 activation and function during DNA repair remain unclear. In this light, we explored SMYD3 in GC cells, focusing on its interaction with key HR proteins, which led us to uncover a previously unrecognized role in chromatin remodeling during DSB repair, and evaluated how its inhibition, alone or with PARPis, disrupts DNA repair and sensitizes tumors to DNA-damaging therapies.

## Methods

### Cell lines

AGS, NCI-N87, KATOIII, HGC-27, HEK293T, HT29 and HCT116 were purchased from ATCC. AGS, NCI-N87, and KATOIII cell lines were maintained in RPMI high glucose without pyruvate (21875-034, Gibco) supplemented with 10% fetal bovine serum (FBS) (A5256701, Gibco) and 1% antibiotics (15140-122, Gibco). The HGC-27, HT29, and HCT116 cell lines were maintained in DMEM high glucose without pyruvate (41965-039, Gibco) supplemented with 10% FBS (A5256701, Gibco) and 1% antibiotics (15140-122, Gibco). HEK293T cells were supplemented with 1% pyruvate (#11360070, Gibco) and 1% non-essential amino acids (NEAA) (#11140, Sigma-Aldrich).

The HGC-27 olaparib-resistant (HGC-27-OLA-R) cell line was established from the HGC-27 parental cell line by continuous exposure to olaparib with stepwise increasing concentrations ranging from 1 µM to 10 µM over approximately 6 months.

To generate the ER-AsiSI-AGS cell line, the pBABE-HA-AsiSI-ER plasmid (a kind gift from Gaelle Legube) was transfected into HEK293T cells that express the structural components needed for retrovirus packaging. Viral supernatants were collected and filtered after transfection, then used to infect AGS cells. Selection was carried out using 0.25 mg/ml puromycin. Cells that survived puromycin selection were expanded and kept under the same selection conditions. Isolated individual clones were further validated through immunofluorescence. Cells were treated with 4-hydroxytamoxifen (4OHT, 2 µM) for 6 h to induce nuclear translocation of ER–AsiSI and site-specific DNA DSBs.

Cells were routinely propagated under standard conditions. All cell lines were tested multiple times throughout the study to ensure they were mycoplasma-free (117048, Minerva Biolabs). All cell cultures were performed in a 37 °C incubator with 5% CO_2_.

### Cell transfection and RNA interference

HCT116-SMYD3-KO and KATOIII cell lines were transiently transfected with mammalian expression plasmids p3xFLAG_CMV14_EMPTY (E7908, Sigma-Aldrich), p3xFLAG_CMV14_SMYD3_WT, or p3xFLAG_CMV14_SMYD3_T22A using Lipofectamine 3000 (L3000015, Thermo Fisher Scientific) according to the manufacturer’s instructions. The plasmids described in the manuscript were generated with specific primers, as previously described [17]. Site-directed mutagenesis was performed using the Q5^®^ Site-Directed Mutagenesis Kit (#E05545, New England Biolabs) according to the manufacturer’s instructions. The primer sequences used for the p3xFLAG-CMV14-SMYD3-WT plasmid were reported previously [17]. Site-directed mutagenesis for the p3xFLAG-CMV14-SMYD3-T22A plasmid was performed using the following primers: P-SDM_SMYD3_T22A_FW 5’-GCGCGCCGTGGCCCCGCTGCG and P-SDM_SMYD3_T22A_RV 5’-AGCCCGTTTCCCCTGTTGGCGGTTGC. For RNA interference, AGS cells were transfected with 5 nM validated siRNAs directed against SMYD3 (ID 34863, Life Technologies) using the HiPerfect reagent (301704, Qiagen) or Lipofectamine 3000 (L3000015, Thermo Fisher Scientific) according to the manufacturer’s instructions. Silencer™ Select Negative Control (4390843, Life Technologies) was used as a control.

### CRISPR/Cas9 system

To generate HCT116-SMYD3-KO cell line the TrueCut Cas9 Protein V2 and SMYD3 TrueGuide gRNA (CRISPR1032607, CRISPR1032618, Thermo Fisher Scientific) were transfected into the HCT116 cell line using Lipofectamine CRISPRMAX Transfection Reagent (CMAX00001, Thermo Fisher Scientific) according to the manufacturer’s instructions. After 48 h, clonal populations of HCT116 cells were isolated using agarose-based cloning rings (C1059, Sigma-Aldrich, Merck). Cell clones were tested for site-specific loss-of-function alterations by PCR, using SMYD3 gRNA sequencing FW 5’-AGCCCGTGAGAC GCCCGCTGCTGG and SMYD3 gRNA sequencing RV 5’-GAAAAGTTCGCAACCGCCAA primers. Sequencing products were purified using the Dye Ex 2.0 Spin Kit (63204, Qiagen) and sequenced on an ABI PRISM 310 Genetic Analyzer (Applied Biosystems).

### Tumorspheres

24-well ultra-low-attachment plates (3473, Corning) were used to culture AGS, NCI-N87, and HGC-27-OLA-R tumorspheres. Three-dimensional (3D) cultures were maintained in DMEM/F12 Advanced (12634010, Gibco) supplemented with 6 mg/mL Glucose (G8769, Sigma-Aldrich, Merck), 2 mM L-Glutamine (25030081, Gibco), 10 ng/mL bFGF (F0291, Sigma-Aldrich, Merck), 20 ng/mL EGF (E9644, Sigma-Aldrich, Merck), B27 supplement (12587010, Gibco), and N-2 supplement (17502048, Gibco). An inverted phase-contrast microscope was used to observe the morphology and growth of tumorspheres.

### Patient-derived organoids

Patient-derived organoids (PDOs) were generated from patient biopsies using the IntestiCult™-SF Organoid Growth Medium Human (100–0340, StemCell Technologies). Briefly, 4 × 10^3^ dissociated gastric glands by Gentle Cell Dissociation Reagent (100–0485, StemCell Technology) were mixed into a DMEM/F-12 (31330038, Gibco) and Matrigel Matrix (356234, Corning) solution at a 1:1 volume ratio in 24-well TC-treated plates (3526, Corning) at 37 °C until Matrigel solidification. Then, 750 µL of IntestiCult™-SF Organoid Growth Medium Human (100–0340, Stemcell Technologies) was added on top of the Matrigel dome. PDOs were passaged every 7–10 days.

### Chemicals

BCI-121 (SML1817), Neocarzinostatin (N9162), 4-Hydroxytamoxifen (H7904), and Trypan blue (T8154) were all purchased from Sigma-Aldrich. EM127 was obtained through the straightforward procedure described in [18]. KU60019 (S1570) and olaparib (S1060) were purchased from SelleckChem. Rucaparib (HY-10617 A) was purchased from MedChemExpress. For each chemical, doses and treatment duration are indicated in the figure legends.

### Cellular assays

Cell proliferation was assessed using the CellTiter 96 AQueous One Solution Cell Proliferation Assay Kit (G3582, Promega), following the manufacturer’s instructions. Cells were seeded in 96-well plates one day before treatment. AGS and NCI-N87 cell lines were treated with EM127 (5 µM), alone or in combination with olaparib (10 µM) or rucaparib (5 µM), for 72 h, as indicated. HGC-27 parental and HGC-27-OLA-R cells were treated with increasing concentrations of olaparib (0–20 µM) for 72 h. KATOIII cells, either untransfected or transiently expressing SMYD3-WT or SMYD3-T22A, were treated with neocarzinostatin (1 nM) for 72 h before cell proliferation analysis.

Cell viability was evaluated using the CellTiter-Glo Luminescent Cell Viability Assay Kit (G7570, Promega), according to the manufacturer’s instructions. Cells were seeded in 96-well plates one day before treatment and exposed to increasing concentrations of BCI-121 (0, 10, 30, 60, 100 µM) or EM127 (0, 0.5, 1, 2.5, 5 µM) for 72 h.

For both assays, reagents were added directly to the culture wells and incubated at 37 °C in a humidified incubator for up to 1 h. The signal was measured using a SPECTROstar Omega microplate reader (BMG Labtech). The proliferation index was calculated as the ratio of treated-cell absorbance to control-cell absorbance.

Cell death was assessed by Trypan blue count. Cells were treated for 72 h under two experimental conditions: either with BCI-121 (100 µM), alone or in combination with olaparib (10 µM), or with the SMYD3 inhibitor EM127 (5 µM), alone or in combination with the PARPis olaparib (10 µM) or rucaparib (5 µM). Control cells were treated with vehicle under the same conditions. Briefly, supernatants (containing dead/floating cells) were collected. Cell pellets were resuspended in 1X PBS, and 10 µl were mixed with an equal volume of 0.01% trypan blue solution (T8154). Viable cells (unstained, trypan blue-negative cells) and dead cells (stained, trypan blue-positive cells) were counted with a phase-contrast microscope, and the percentage of dead cells was calculated.

For live/dead assays, tumorspheres and patient-derived tumor organoids (PDTOs) were treated or not with EM127 (5 µM), alone or in combination with olaparib (10 µM) or rucaparib (5 µM), for 72 h. After treatments, tumorspheres and PDTOs were stained using the LIVE/DEAD^®^ Cell Imaging Kit (R37601, Thermo Fisher Scientific) according to the manufacturer’s instructions. ZEN microscopy software was used to quantify the average fluorescence brightness. Digital image acquisition was assessed on a Zeiss Axio Observer fluorescence microscope using a 10x magnification objective.

### Chromatin immunoprecipitation

Chromatin isolated from ER-AsiSI-AGS cells was subjected to chromatin immunoprecipitation (ChIP) using the MAGnify Chromatin Immunoprecipitation Kit (492024, Thermo Fisher Scientific) according to the manufacturer’s instructions. Briefly, cells were cross-linked in 1% formaldehyde (252549, Sigma-Aldrich) for 10 min. Cross-linking was blocked with 0.125 M glycine (23390.04, Serva) for 5 min, then cells were washed with 1X PBS and lysed in lysis buffer. Chromatin was sonicated to a fragment length of about 200–500 bp and immunoprecipitated with 1 µg of following antibodies: anti-SMYD3 (ab187149, Abcam), anti-H4K20me2 (ab9052, Abcam), anti-H3K9me3 (ab8898, Abcam), anti-H4K16ac (ab109463, Abcam), anti-H3K79me2 (ab3594, Abcam), anti-53BP1 (NB100-304, Novus), anti-RAD50 (3427, Cell Signaling Technology), anti-ATM (2873, Cell Signaling Technology), anti-CHK2 (6334, Cell Signaling Technology), anti-BRCA1 (14823, Cell Signaling Technology), anti-RPA32/RPA2 (35869, Cell Signaling Technology), anti-BRCA2 (ab123491, Abcam), and anti-RAD51 (8875, Cell Signaling Technology). IgG antibodies were used as negative controls. Quantitative real-time PCR was performed using PowerUp SYBR Green Master Mix (A25741, Thermo Fisher Scientific). ChIP primer sequences (DSB1, DSB2, DSB3, and NO DSB) are available upon request.

### Co-immunoprecipitation

Cells were lysed either in lysis buffer (50 mM Tris-HCl, pH 7.4, 5 mM EDTA, 250 mM NaCl, and 1% Triton X-100) supplemented with protease and phosphatase inhibitors (78443, Thermo Fisher Scientific), or using the Nuclear Extraction Kit (ab13474, Abcam), according to the manufacturer’s instructions, to obtain whole-cell lysates or nuclear fractions, respectively. In both cases, 10% of the input was retained as a loading control. For immunoprecipitation, 1 µg of antibody was pre-coupled to Dynabeads Protein A or G (10002D or 10004D, Thermo Fisher Scientific) in 100 µl of 0.01% Tween-20 in PBS for 45 min at room temperature on a rocking platform. Lysates or nuclear fractions were then incubated with antibody–bead complexes for 1 h at room temperature with rotation. Immunocomplexes were extensively washed, eluted by boiling in Laemmli sample buffer (1610747, Bio-Rad Laboratories), and analyzed by SDS-PAGE and immunoblotting. IgG antibodies were used as negative controls. Primary antibodies: anti-SMYD3 (12859, Cell Signaling Technology), anti-RAD50 (3427, Cell Signaling Technology), anti-ATM (2873, Cell Signaling Technology), anti-phospho-ATM (5883, Cell Signaling Technology), anti-CHK2 (6334, Cell Signaling Technology), anti-BRCA1 (14823, Cell Signaling Technology), anti-RPA32/RPA2 (35869, Cell Signaling Technology), anti-PALB2 (ab202970, Abcam), anti-BRCA2 (ab123491, Abcam), anti-RAD51 (8875, Cell Signaling Technology), anti-phospho-SMYD3 (custom made, code 4352, Thermo Fisher Scientific), and anti-FLAG (F1804, Sigma-Aldrich). Rabbit IgG HRP (NA934V, GE Healthcare) was used as a secondary antibody and revealed using the ECL-plus chemiluminescence reagent (RPN2232, GE Healthcare).

### Droplet digital PCR (ddPCR) assay

ddPCR was performed using 10 ng of RNA extracted from tissue samples and PDOs. RNA was extracted using the Purelink RNA Micro Kit (12183016, Thermo Fisher Scientific) and retro-transcribed to cDNA using the Super-Script VILO cDNA Synthesis Kit (11755050, Thermo Fisher Scientific) according to the manufacturer’s instructions. Reactions were prepared using the ddPCR EvaGreen Supermix (1864033, Bio-Rad Laboratories) and specific primers according to the manufacturer’s instructions. Droplets were processed as described above and amplified under the following cycling protocol: a denaturation step at 95 °C for 5 min, followed by 40 cycles at 95 °C for 30 s and 60 °C for 1 min, a post-cycling step for signal stabilization at 4 °C for 5 min and 95 °C for 5 min, and an infinite hold at 4 °C. The plate was then transferred into a QX200 Reader (Bio-Rad Laboratories). Data were analyzed using Bio-Rad QX Manager 1.2 Standard Edition. ddPCR primer sequences (EpCAM, CDH1, and SMYD3) are available upon request.

### Immunoblotting

Whole-cell extracts were obtained from cells and tissues. Cells were collected and homogenized in lysis buffer (50mM Tris-HCl, pH 7.4, 5mM EDTA, 250mM NaCl, and 1% Triton X-100) supplemented with protease and phosphatase inhibitors (78443, Thermo Fisher Scientific). To validate the phospho-specific signal, whole-cell lysates were prepared in lysis buffer supplemented with protease inhibitors but lacking phosphatase inhibitors. Each lysate was divided into two fractions. One fraction was used without further treatment, whereas the other was incubated with 60 U of recombinant shrimp alkaline phosphatase (rSAP; M0371L, New England Biolabs) in 1× rCutSmart Buffer (B6004S, New England Biolabs) at 37 °C for 2 h before immunoblot analysis. Tissues were collected and homogenized in T-PER™ Tissue Protein Extraction Reagent (78510, Thermo Fisher Scientific) supplemented with protease and phosphatase inhibitors (78443, Thermo Fisher Scientific). Tissue dissociation was carried out using the gentleMACS Tissue Dissociator (130-093-235, Miltenyi Biotec) and gentleMACS™ M Tubes (130-093-236, Miltenyi Biotec). 30 µg of protein extract from each sample was denatured in 4x Laemmli sample buffer (1610747, Bio-Rad Laboratories) and loaded onto an SDS-polyacrylamide gel for immunoblot analysis. Primary antibodies used: anti-β-ACTIN (3700, Cell Signaling Technology), anti-cleaved PARP (5625, Cell Signaling Technology), anti-phospho-SMYD3 (custom made, code 4352, Thermo Fisher Scientific), anti-GAPDH (5174, Cell Signaling Technology), anti-KLF4 (12173, Cell Signaling Technology), anti-OCT4 (2750, Cell Signaling Technology), anti-VINCULIN (13901, Cell Signaling Technology), and anti-SMYD3 (12859, Cell Signaling Technology). Rabbit IgG HRP and Mouse IgG HRP (1706515 and 1706516, respectively, Bio-Rad Laboratories) were used as secondary antibodies and were revealed using the Clarity Max Western ECL Substrate (1705062, Bio-Rad Laboratories).

### *In silico* P-tripeptides screening

The screening of tripeptides P1-P19 was conducted according to methods outlined in previous publications [8, 17, 19]. In particular, we utilized a library of tripeptides (P1-P19) to assess their in vitro binding affinity to SMYD3. Subsequently, we employed these tripeptides as in silico probes to identify human proteins (termed P-proteins) that contain these specific P-tripeptides. To identify all human proteins encompassing the P1–P19 tripeptide as potential candidates for SMYD3 interaction, the human proteome was screened using the UniProt Peptide Search tool (https://www.uniprot.org/peptidesearch). A total of 169,671 human proteins reported in the UniProt database at the time of the analysis (December 2018) were searched and mapped for each P-tripeptide, and 8,650 proteins (reviewed; UniProt annotation score = 5) were identified to contain at least one P-tripeptide.

### In silico clustering of the P-proteins involved in DNA repair-related processes

In silico clustering of the P-proteins involved in DNA repair was confirmed by evaluating the relevant Reactome cluster (DNA Repair, Reactome ID: R-HSA-73894.3) (https://reactome.org). At the time of analysis (January 2022), 147 P-proteins were identified as effectors of different DNA repair-related pathways [8], and 35 P-proteins were specifically involved in the HR pathway (HDR through Homologous Recombination (HRR); Reactome ID: R-HSA-5685942.4). Thus, we analyzed in silico all 35 P-proteins, describing them for the P-tripeptide matches and positions, gene names, protein names, lengths, functions, and Reactome IDs supplied in the UniProt database. We further validated *in cellulo* the ability of selected candidates to interact with SMYD3 based on their oncogenic relevance.

### In silico prediction analysis of SMYD3 relative surface accessibility

We evaluate the relative surface accessibility of Threonine 22 using an in silico prediction analysis with the NetSurfP-3.0 server (https://services.healthtech.dtu.dk/services/NetSurfP-3.0/). The NetSurfP-3.0 server provides predictions for the surface accessibility, secondary structure, disorder, and phi/psi dihedral angles of amino acids in a given amino acid sequence.

### In silico prediction analysis of SMYD3 phosphorylation

In silico prediction analysis was performed using NetPhos-3.1 (https://services.healthtech.dtu.dk/services/NetPhos-3.1/?utm), Phosphosite Plus (https://www.phosphosite.org/?utm), GPS 5.0 (https://gps.biocuckoo.cn/), and Phosida (http://www.phosida.com/) servers.

### Mass spectrometry analysis

Mass spectrometry analysis was conducted by the Cogentech SRL service. Gel bands underwent reduction using dithiothreitol (DTT) (A39255, Thermo Fisher Scientific), alkylation using iodoacetamide (IAA) (A39271, Thermo Fisher Scientific), and double enzymatic digestion using endoproteinases AspN (V1621, Promega) which selectively cleaves protein and peptide bonds N-terminal to aspartic acid residues and Glu-C (V1651, Promega) that specifically cleaves at the C-terminus of either aspartic or glutamic acid residues. Then, the flow-through was treated with C18 Spin Tips & Columns (84850, Thermo Fisher Scientific) for desalting. The samples and the desalted flow-through were further purified with SP3 and then analyzed by nLC-ESI-MS/MS on a Q Exactive HF mass spectrometer (Thermo Fisher Scientific) with a 45 min gradient. Samples were run in technical duplicate, in a positive mode with electrospray ionization. Data acquisition and processing were performed with Analyst TF (version 1.7.1, AB SCIEX). Data were analyzed using the Proteome Discoverer, Mascot, and Scaffold software. The parameter settings of data processing were as follows: Database = Uniprot_CP_Human_2020 (Database Uniprot_cp_Human SMYD3; Human sequence, Accession Numbers: Q9H7B4); Modifications = Acetyl (Protein N-term), Carbamidomethyl (C), Oxidation (M), Phosphorylation (STY); Peptide Thresholds: 95.0% minimum; Protein Thresholds: 99.0% minimum; 2 peptides/protein minimum.

### Quantitative real-time PCR

PCR assays were performed in 96-well optical reaction plates using a QuantStudio 3 machine (Thermo Fisher Scientific). Each assay was carried out in triplicate wells. Baseline values of amplification plots were set automatically, and threshold values were kept constant to obtain normalized cycle times and linear regression data. The following reaction mixture per well was used: 5 µL of PowerUp SYBR Green Master Mix (A25741, Thermo Fisher Scientific), 1.1 µL of primers at a final concentration of 100 nM, 2.4 µL of RNase-free water, and 1.5 µL of cDNA. For all experiments, the following PCR conditions were used: denaturation at 95 °C for 30 s, followed by 40 cycles at 95 °C for 15 s and 60 °C for 30 s. Relative quantification was done using the ddCT method.

### Analysis of *SMYD3* expression and clinically relevant genomic alterations in TCGA-STAD

To investigate the association between SMYD3 mRNA expression and clinically actionable genomic alterations, we analyzed 407 stomach adenocarcinoma (STAD) samples from The Cancer Genome Atlas (TCGA) Pan-Cancer Atlas cohort, accessed through the cBioPortal database (https://www.cbioportal.org, accessed on January 2025). *SMYD3* mRNA expression levels were quantified as Z-scores, and tumors were stratified into *SMYD3*-high (> median; *n* = 202) and *SMYD3*-low (≤ median; *n* = 205) groups. Genomic alterations were annotated using the OncoKB precision oncology database (https://www.oncokb.org, accessed on January 2025). Clinically actionable alterations (OncoKB Levels 1–3) were assessed in 23 DNA damage response (DDR) genes (*ATM*,* ATR*,* BARD1*,* BRCA1*,* BRCA2*,* BRIP1*,* CHEK1*,* CHEK2*,* FANCA*,* FANCL*,* IDH1*,* MLH1*,* MRE11A*,* NBN*,* PALB2*,* PTEN*,* RAD51B*,* RAD51C*,* RAD54L*,* POLD1*,* POLE*,* ERCC2*, and *SMARCA4*). In addition, clinically relevant genomic alterations in STAD, including microsatellite instability-high (MSI-H), high tumor mutation burden (TMB-H), *ERBB2* amplification, *EGFR* amplification, *MTAP* deletion, *BRAF* V600E, *KRAS* G12C, and *TP53* hotspot mutations, were also evaluated. Co-occurrence and mutual exclusivity between *SMYD3* mRNA overexpression status and clinically actionable alterations were assessed using the “DISCOVER” R package (v 0.9.4) [20]. Briefly, a binary gene-by-sample matrix was generated, where each entry indicates the presence (1) or absence (0) of a somatic gene alteration in the GC tumor. The alteration status of all genes across all tumors for each cancer type was used to generate a null distribution for background alternation rate estimation and to compute the statistical significance of co-occurrence and pairwise mutual exclusivity.

### Analysis of *SMYD3* expression across GC cell lines

We analyzed endogenous *SMYD3* mRNA and protein expression levels across the panel of human gastric cancer cell lines available from the DepMap portal (https://depmap.org/portal/; Public 26Q1 release, accessed on 24 June 2026) [21]. For these cell lines, *SMYD3* mRNA expression was retrieved as log2-transformed transcript abundance (log2[TPM + 1]; *n* = 41 gastric cell lines), and SMYD3 protein expression as normalized mass-spectrometry protein abundance (log2 ratio; *n* = 14 gastric cell lines with available proteomic data) [21, 22]. Cell lines were ranked by *SMYD3* expression, and the SMYD3-low reference group was defined as the lowest-expressing quartile of gastric cell lines. Cell lines expressing *SMYD3* above this reference were classified as higher-expressing *SMYD3*. The fold-change was computed as the difference between the median log2 *SMYD3* value of the cell lines and the median log2 SMYD3 value of the corresponding reference group, then exponentiated (2^log2FC) to give the linear fold-change [23]. Analyses were performed in R version 2025.09.1.

### Immunofluorescence and foci counting

Cells were seeded on glass coverslips, treated as indicated for each experiment, and then fixed with 4% paraformaldehyde and permeabilized using 0.1% Triton X-100. Coverslips were incubated with the indicated primary antibodies and then with Alexa Fluor 488 (A-11094, Thermo Fisher Scientific) and 647 (A-32728, Thermo Fisher Scientific) secondary antibodies; nuclei were counterstained using DAPI (D9542, Sigma-Aldrich, Merck). Slides were sealed using ProLong Diamond Antifade Mountant (P36961, Thermo Fisher Scientific). Images were acquired using a Zeiss fluorescence microscope. Primary antibodies: anti-SMYD3 (ab183498, Abcam), anti-HA-Tag (SAB2702196, Sigma-Aldrich), and anti-γH2AX (9718, Cell Signaling Technology; 05-636, Sigma-Aldrich Millipore). Densitometric evaluation was performed using ZEN microscopy software. Foci were scored by fluorescence microscopy using a 63X magnification objective and digital image acquisition on a Zeiss Axio Observer fluorescence microscope.

### DR-GFP homologous recombination assay

AGS cells were seeded into 24-well plates and transfected with 500 ng of the I-SceI expression plasmid (pCBASce) and 500 ng of the DR-GFP reporter plasmid using Lipofectamine 3000 transfection reagent (Life Technologies). For SMYD3 depletion or pharmacological inhibition, cells were transfected with 30 nM SMYD3-targeting siRNA (siRNA ID34863) or pretreated with EM127 (5 µM), respectively, 24 h before I-SceI/DR-GFP transfection. GFP fluorescence, indicative of HR-mediated repair efficiency, was evaluated 48 h after reporter transfection by automated fluorescence microscopy using the Celldiscoverer 7 platform. Cells were counterstained with Hoechst 33342 for nuclear visualization and normalization of GFP-positive cells.

### Luciferase NHEJ repair reporter assay

The pGL3 promoter vector (212939, Promega) was linearized using the HindIII enzyme (R3104, New England Biolabs) to induce DSBs. AGS cells were subjected to SMYD3 inhibition either pharmacologically, through pre-treatment with EM127, or genetically, through transfection with SMYD3-targeting siRNA (siRNA ID34863) for 24 h. Cells were then transfected with 500 ng of either the linearized plasmid or the uncut pGL3 control plasmid using Lipofectamine 3000 (L3000015, Thermo Fisher Scientific), along with 10 ng of the Renilla luciferase vector as a transfection efficiency control. Luciferase activity was assessed 24 h post-transfection using the Dual Luciferase Reporter Assay Kit (E1910, Promega). Firefly luciferase activity in each sample was normalized to the Renilla luciferase signal. The percent reactivation of NHEJ was calculated by normalizing the linearized pGL3 signal to the uncut pGL3 control signal. Data are presented as relative repair efficiencies, in which the proportion of reactivation in the experimental condition (EM127 treatment) is normalized to the control condition.

### Statistical analysis

The statistical significance of the results was analyzed using Student‘s t-test, and *p < 0.05 was considered statistically significant.

## Results

### SMYD3 defines a subset of GCs lacking actionable DDR alterations and interacts with HR repair proteins

**Fig. 1:**
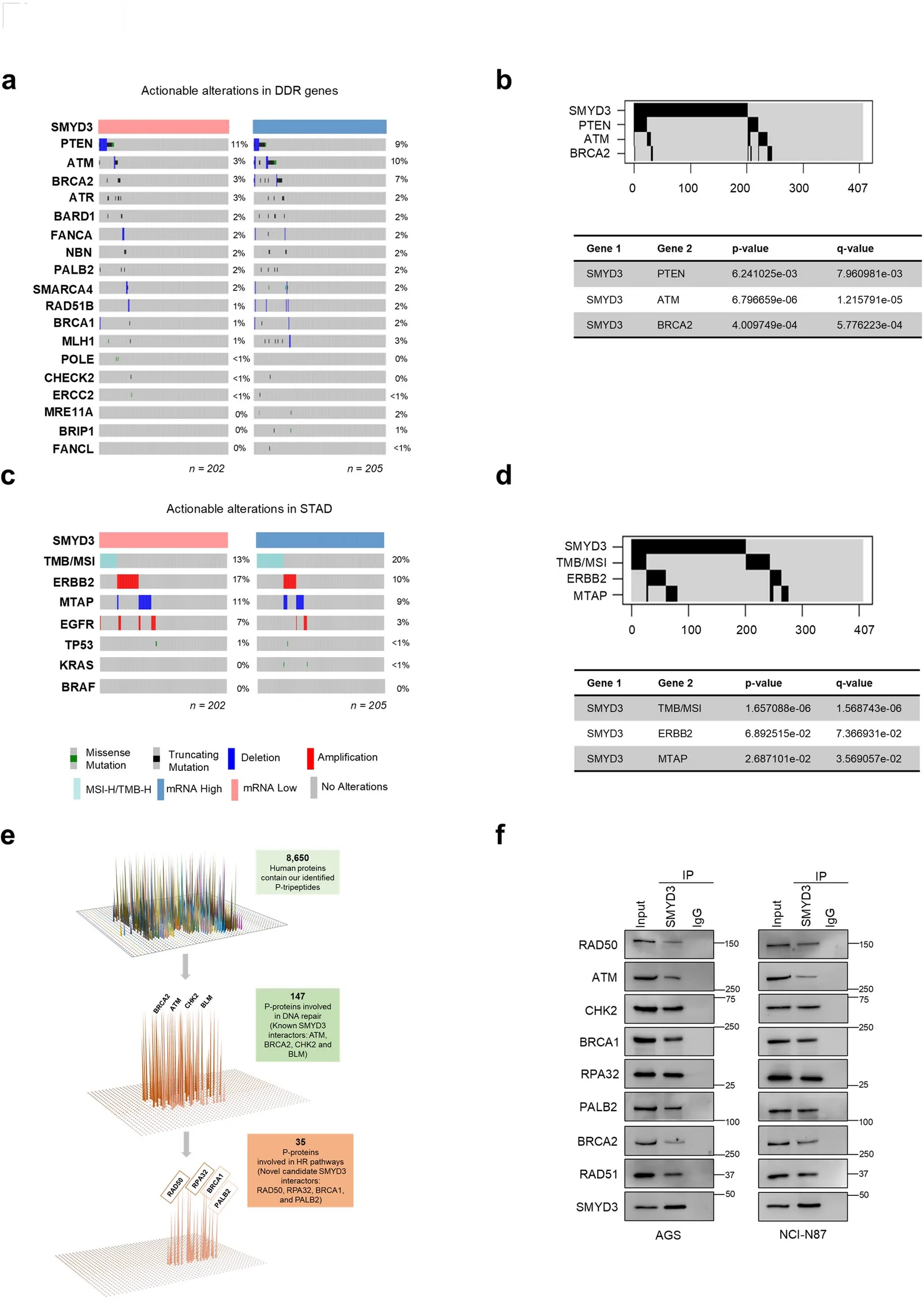
SMYD3 characterizes GCs lacking actionable DDR alterations and is linked to HR repair machinery.** a** Oncoprint showing *SMYD3* mRNA expression status and actionable alterations in DDR genes across 407 TCGA Pan-Cancer Atlas Stomach adenocarcinoma (STAD) tumors. Columns represent distinct tumors, and rows indicate genes. STAD tumors are classified into SMYD3-high (*n* = 202) and SMYD3-low (*n* = 205) groups. Gray boxes indicate absence of alterations, whereas colored annotations denote mutation types (missense, truncating), copy number alterations (amplification, deletion). The frequency of alterations for each gene is reported on the right for SMYD3-high and SMYD3-low tumors. **b** Mutual exclusivity analysis between SMYD3 overexpression and actionable DDR gene alterations (*PTEN, ATM, BRCA2*) was performed using the DISCOVER algorithm. Tumors harboring actionable alterations are highlighted in black, whereas those without such alterations are shown in gray. Statistical significance was assessed using pairwise DISCOVER testing, and corresponding p-values and q-values are reported below. **c** Oncoprint of SMYD3 expression and clinically actionable alterations relevant to STAD, including MSI-H/TMB-H status, *ERBB2* amplification, *MTAP* deletion, *EGFR* amplification, *TP53*, *KRAS*, and *BRAF* mutations. Annotation scheme and visualization are as described in **(a)**. The frequency of alterations for each gene is reported on the right for SMYD3-high (*n* = 202) and SMYD3-low (*n* = 205) tumors. **d** Mutual exclusivity analysis between SMYD3 overexpression and clinically actionable alterations relevant to STAD, including MSI/TMB status, *ERBB2*, and *MTAP*, using the DISCOVER framework. The corresponding p-values and q-values are reported below. **e** Procedural scheme of the *in silico* P-tripeptides screening. Proteins were clustered based on their functional involvement as annotated in the corresponding Uniprot entry, and the clustering was confirmed in the Reactome database. **f** Co-IP analysis of endogenous SMYD3 and key HR proteins in the AGS and NCI-N87 GC cell lines. Immunoprecipitation was performed using an anti-SMYD3 antibody, and IgG was used as a negative control. Data are representative of at least three independent experiments

To investigate the association between SMYD3 expression and clinically actionable genomic alterations, we analyzed 407 stomach adenocarcinoma (STAD) samples with available gene expression and mutation data from The Cancer Genome Atlas (TCGA) Pan-Cancer Atlas cohort, accessed via the cBioPortal database. Tumors were stratified based on *SMYD3* mRNA z-scores into SMYD3-high (> median; *n* = 202) and SMYD3-low (≤ median; *n* = 205). To evaluate the therapeutic landscape, mutational profiles were annotated using the OncoKB precision oncology database. Clinically actionable alterations (OncoKB Levels 1–3) were examined in 23 DNA damage response (DDR) genes. Additionally, we examined actionable alterations relevant to STAD, such as high microsatellite instability (MSI-H), high tumor mutation burden (TMB-H), *ERBB2* amplification, *EGFR* amplification, *MTAP* deletion, *BRAF* V600E, *KRAS* G12C, and *TP53* Y200C mutations. In the SMYD3-high group, 52 out of 202 tumors (25.7%) had actionable DDR alterations involving 15 genes (*ATM*,* ATR*,* BARD1*,* BRCA1*,* BRCA2*,* CHEK2*,* FANCA*,* MLH1*,* NBN*,* PALB2*,* PTEN*,* RAD51B*,* POLE*,* ERCC2*, and *SMARCA4*) (Fig. 1a). Notably, co-occurrence and mutual exclusivity analysis showed significant mutual exclusivity between SMYD3-high status and alterations in *PTEN*,* ATM*, and *BRCA2*, with no significant co-occurring alterations identified (Fig. 1b). Furthermore, 92 out of 202 (45.5%) SMYD3-high tumors displayed actionable alterations relevant to STAD, while 110/202 (54.5%) lacked such alterations (Fig. 1c). Importantly, mutual exclusivity analysis also revealed significant mutual exclusivity between SMYD3-high status and *ERBB2* amplification, MSI-H/TMB-H, and *MTAP* deletion, with no significant co-occurrences (Fig. 1d). These findings suggest that SMYD3-high tumors may constitute a subgroup lacking canonical actionable alterations, emphasizing the importance of further research.

To validate these findings, we analyzed endogenous SMYD3 expression levels across a panel of human GC cell lines available in DepMap (https://depmap.org/portal/; Public 26Q1 release, accessed 24 June 2026) [21], comprising 41 cell lines with transcriptomic and 14 with proteomic data. The lowest SMYD3-expressing quartile was used as the SMYD3-low reference group. Relative to the median of the SMYD3-low reference group, AGS, HGC-27, and NCI-N87 cells showed 4.4-, 2.8-, and 1.6-fold higher *SMYD3* mRNA levels and 4.8-, 5.8-, and 1.6-fold higher SMYD3 protein levels, respectively. In contrast, KATOIII cells showed the lowest SMYD3 expression of all GC lines at both the mRNA and protein levels (Supplementary Fig. 1a–b). Remarkably, AGS cells ranked among the highest SMYD3-expressing gastric cell lines at both the mRNA and protein levels. To validate the DepMap-based expression profile, we analyzed endogenous SMYD3 protein levels in the selected GC cell lines (Supplementary Fig. 1c). As positive controls for high SMYD3 expression, we included the CRC cell lines HCT116 and HT29, which have previously been characterized as SMYD3-high models [24]. Based on these results, AGS cells were selected as the primary model for subsequent mechanistic studies.

Next, we characterized somatic mutations and copy-number alterations in key HR-related genes, retrieved from the Catalog of Somatic Mutations in Cancer (COSMIC) database, to assess the mutational status of the HR pathway in GC cell lines. This analysis identified AGS and NCI-N87 as HR wild-type (WT) (Supplementary Fig. 1d), whereas the HGC-27 cell line was classified as HR-compromised, supported by evidence of BRCA2-related defects and sensitivity to PARPis [25, 26].

Recently, taking advantage of a library of rare P-tripeptides (collectively termed P1-P19 tripeptides) tested for their in vitro binding affinity to SMYD3 by Surface Plasmon Resonance (SPR) analysis, we performed an in silico tripeptide screening of the human proteome using each tripeptide as a sequence probe to identify a total of 8,650 proteins (termed P-proteins) encompassing at least one P-tripeptide [8, 17]. Briefly, each P-tripeptide was used as a sequence probe to screen the human proteome and identify proteins containing identical tripeptide motifs. The rationale underlying this approach is that proteins sharing sequence motifs previously validated as SMYD3-binding elements may represent candidate SMYD3 interactors. Among 8,650 P-proteins, in our previous reports, we identified BRCA2, ATM, CHK2, MTOR, BLM, MET, AMPK, p130, EP300, TRRAP, and c-MYC as direct interactors of SMYD3 [8, 15, 17, 19]. To investigate the potential involvement of SMYD3 in the DNA damage response, we subsequently focused on the subset of P-proteins annotated in UniProt and Reactome as DNA repair factors. Interestingly, 147 P-proteins were involved in DNA repair and were subsequently grouped based on UniProt annotations, published literature, and Reactome pathway analyses. Specifically, within the DNA repair P-proteins cluster, the HR subset included 35 candidate SMYD3-interacting proteins (Supplementary Table 1), from which those most significantly associated with GC tumorigenesis were identified as potential novel interactors (Fig. 1e). Therefore, we evaluated the direct interaction of SMYD3 with key HR proteins by performing co-immunoprecipitation (Co-IP) assays with anti-SMYD3 antibody in total cell lysates from AGS and NCI-N87 cell lines (Fig. 1f). We observed that SMYD3 co-immunoprecipitated with RAD50, ATM, CHK2, BRCA1, RPA32, PALB2, BRCA2, and RAD51, suggesting that it interacts with the HR complex and may contribute to its function. These interactions were further confirmed by reverse Co-IP (Supplementary Fig. 1e). These findings revealed that SMYD3 interacts with HR proteins that exert their functions at multiple stages of repair, suggesting a role across the entire HR repair process.

### Role of SMYD3 in coordinating histone modifications and DDR factor recruitment at DSBs

The observed interactions between SMYD3 and HR proteins, which involved multiple stages of the DDR, suggested that its histone methyltransferase activity may facilitate chromatin remodeling at DSBs and promote the recruitment of HR factors. To investigate the role of SMYD3 at DSBs, we generated an inducible ER–AsiSI AGS cell line (ER–AsiSI–AGS), in which 4-hydroxytamoxifen (4OHT) triggers nuclear localization of the AsiSI endonuclease to rapidly induce ~ 150 site-specific DSBs across the genome [27] (Supplementary Fig. 2a). Since the HA-tagged AsiSI restriction enzyme is fused to the estrogen receptor (ER), we used immunofluorescence to confirm nuclear localization and γH2AX-marked DSB formation (Supplementary Fig. 2a–b). Two HR-prone AsiSI sites (chr1:88,992,932 and chr22:38,468,089), previously identified among the top 80 AsiSI-induced DSBs [28], were selected for analysis and are hereafter referred to as DSB1 and DSB2 (Supplementary Fig. 2c). A genomic region not targeted by AsiSI was included as a negative control (NO DSB).

**Fig. 2:**
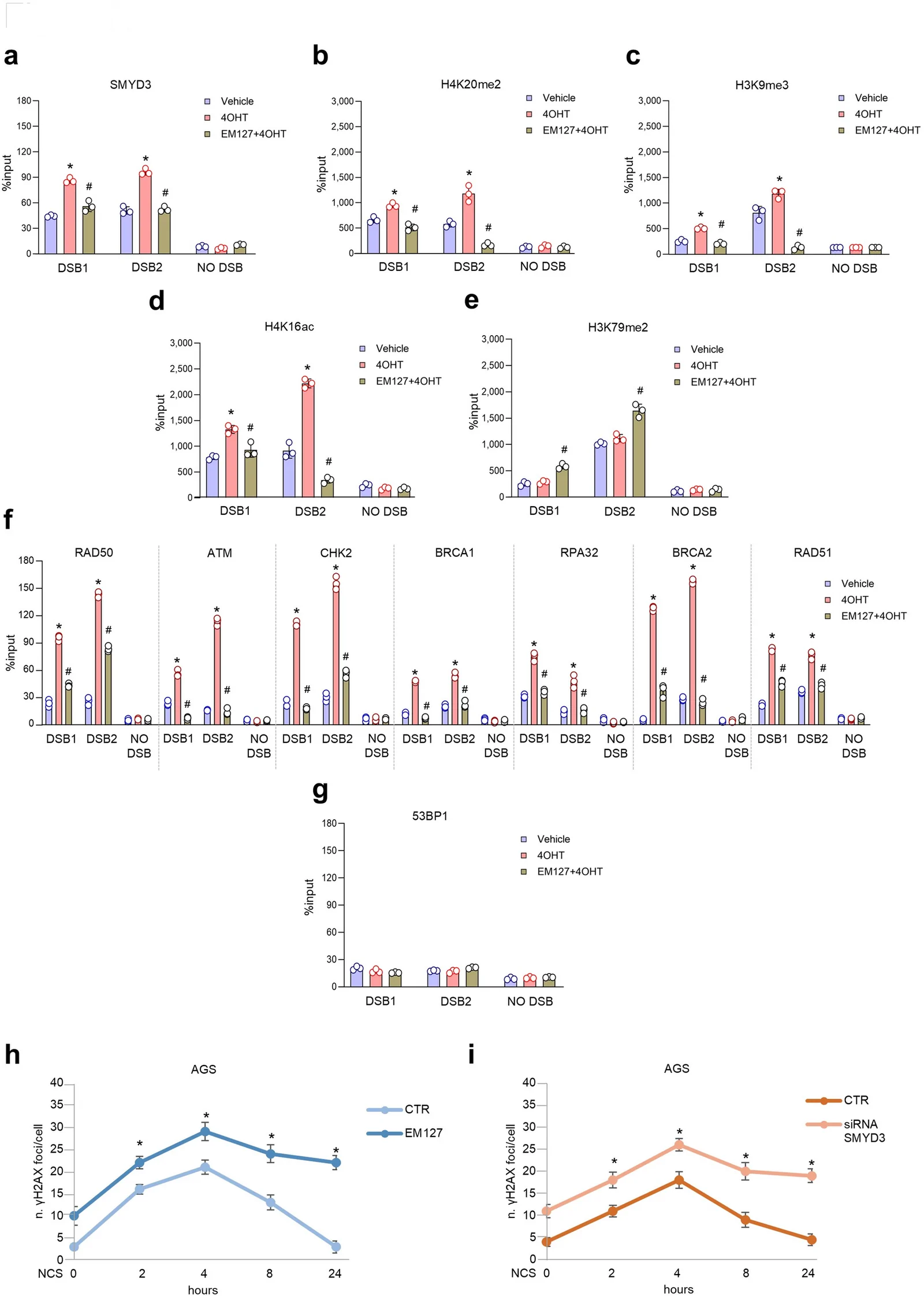
SMYD3 inhibition reshapes chromatin marks and HR proteins recruitment at AsiSI-induced DSB sites.** a** ChIP–qPCR analysis was performed using primer pairs designed within 100 bp of the AsiSI-induced DSB sites (DSB1–2) or at a non-cleaved control locus (NO DSB) in ER-AsiSI-AGS cells, pre-treated or not with the SMYD3 inhibitor EM127 (5 µM, 24 h) and subsequently treated or not with 4OHT (2 µM, 6 h) to induce DSBs. Chromatin was immunoprecipitated using an anti-SMYD3 antibody. **b–e** ChIP–qPCR analysis performed at AsiSI-induced DSB sites (DSB1–2) and at a non-cleaved control locus (NO DSB) under the same experimental conditions as in (**a**), using antibodies anti-H4K20me2 (**b**), anti-H3K9me3 (**c**), anti-H4K16ac (**d**), and anti-H3K79me2 **(e)**, as indicated. **f** ChIP–qPCR analysis at AsiSI-induced DSB sites (DSB1–2) and at a non-cleaved control locus (NO DSB), using antibodies anti-RAD50, anti-ATM, anti-CHK2, anti-BRCA1, anti-RPA32, anti-BRCA2, and anti-RAD51. **g** ChIP–qPCR analysis at AsiSI-induced DSB sites (DSB1–2) and at a non-cleaved control locus (NO DSB), using anti-53BP1 antibody. Quantification was performed using the % input method. Significance was determined by Student’s t-test; *p < 0.05, 4OHT-treated vs. untreated. #*p* < 0.05 combined treatment (EM127 + 4OHT) vs. single treatment (4OHT). Data are representative of at least three independent experiments. **h-i** AGS cells were treated with NCS-induced DNA damage (1 nM) and analyzed at the indicated time points (0, 2, 4, 8, and 24 h). The repair kinetics were evaluated in cells treated with EM127 (5 µM, 24 h) (**h**) or in cells transfected with SMYD3-siRNA (5 nM, 48 h) **(i)**, compared with the corresponding control conditions. Cells were immunostained for γH2AX (green), and nuclei were counterstained with DAPI (blue). The graphs show the quantification of the mean number of γH2AX foci/cell over time. Scale bar: 5 μm. Statistical analysis was determined by Student’s t-test (*p < 0.05)

We first assessed SMYD3 recruitment to DSB sites by chromatin immunoprecipitation (ChIP), revealing its enrichment at HR-prone DSBs upon 4OHT treatment (Fig. 2a). Notably, its recruitment to DNA-damaged sites was markedly affected upon its pharmacological inhibition with the inhibitor EM127 [18] (Fig. 2a). To investigate whether SMYD3 affects histone modifications at DSB sites, we analyzed key histone marks associated with DNA repair. Given that SMYD3 modulates histone H4 lysine 20 dimethylation (H4K20me2) in vitro, a critical mark involved in the early recognition of DNA damage [29], we evaluated its accumulation at DSB sites. ChIP analysis revealed a marked enrichment of H4K20me2 following DSB induction, whereas pharmacological inhibition of SMYD3 with EM127 significantly reduced its accumulation at damaged chromatin (Fig. 2b). We next examined histone H3 lysine 9 trimethylation (H3K9me3), another key chromatin modification involved in DDR [30]. As observed for H4K20me2, H3K9me3 was significantly enriched at DSB sites following 4OHT treatment, while SMYD3 inhibition reduced its accumulation (Fig. 2c). Although SMYD3 has not been reported to methylate H3K9 in vitro [31] directly, these findings suggest that its activity is required for H3K9me3 enrichment at DSB sites, potentially via UHRF1-mediated SUV39H1 recruitment [32, 33]. Because H3K9me3 typically promotes the recruitment of the acetyltransferase TIP60, which catalyzes histone H4 lysine 16 acetylation (H4K16ac) [30], we next examined this downstream mark. Consistent with the reduction in H3K9me3, H4K16ac enrichment at DSBs was likewise significantly diminished upon SMYD3 inhibition (Fig. 2d), confirming the existence of a multi-step chromatin remodeling regulated by SMYD3 during early DDR. We next investigated the histone H3 lysine 79 dimethylation (H3K79me2), a modification known to compensate for H4K20me2 alterations during repair. Unlike H4K20me2 and H3K9me3, H3K79me2 levels remained unchanged following DNA damage induction alone. However, SMYD3 inhibition led to a significant increase in this mark at 4OHT-induced DSBs (Fig. 2e), suggesting the activation of a compensatory chromatin remodeling mechanism that may help preserve DNA repair capacity when SMYD3-dependent signaling is impaired.

To determine how these SMYD3-dependent chromatin changes affect the assembly of DNA repair machineries, we examined the recruitment of key HR proteins and the NHEJ sensor 53BP1 at site-specific DSBs. At HR-prone AsiSI-induced DSBs, we observed a significant enrichment of key HR machinery proteins, including RAD50, ATM, CHK2, BRCA1, RPA32, BRCA2, and RAD51 (Fig. 2f). Pharmacological inhibition of SMYD3 markedly impaired the recruitment of all these proteins, demonstrating that SMYD3 activity is required for the efficient assembly of the HR machinery at transcriptionally active DSBs. Conversely, since 53BP1 is rapidly displaced from resected DNA ends by BRCA1 during HR [34], its recruitment was not detected at these HR-prone AsiSI sites (Fig. 2g). Therefore, to evaluate the role of SMYD3 in 53BP1 dynamics, we analyzed its recruitment at a validated NHEJ-prone DSB locus, referred to as DSB3 (chr6:89,638,094) [28]. At this site, 53BP1 ChIP demonstrated a robust accumulation upon damage induction (Supplementary Fig. 2d). Notably, pharmacological inhibition of SMYD3 significantly decreased 53BP1 recruitment at this NHEJ-prone site, suggesting that SMYD3 may contribute to the recruitment of upstream DDR factors across different DNA repair contexts. Collectively, these findings demonstrate that SMYD3 regulates the remodeling of key histone modifications at DNA damage sites, thereby promoting the efficient recruitment of DDR factors and the proper execution of DNA repair.

### ATM-mediated SMYD3 phosphorylation promotes HR complex assembly in GC

Following our observation that SMYD3 inhibition impairs DDR protein recruitment on damaged chromatin, we next investigated whether SMYD3 contributes to the kinetics of DSB repair. AGS cells were treated with neocarzinostatin (NCS), a DSB-inducing agent that generates breaks randomly, often in heterochromatin, providing distinct chromatin contexts and repair kinetics compared with the AsiSI model [28]. The resolution of DNA damage was monitored over time by immunofluorescence analysis of γH2AX nuclear foci in control cells, as well as in cells treated with the SMYD3 inhibitor EM127 or transfected with SMYD3-specific siRNA. While control cells progressively resolved γH2AX foci following DNA damage, both pharmacological inhibition and genetic depletion of SMYD3 markedly delayed foci resolution, indicating impaired DSB repair kinetics (Fig. 2h-i). Consistent with this observation, analysis performed 24 h after NCS treatment revealed a significantly higher number of γH2AX nuclear foci in SMYD3-inhibited and SMYD3-silenced cells compared with controls (Fig. 2h-i, Supplementary Fig. 2e), indicating the accumulation of unrepaired DSBs and demonstrating that SMYD3 activity is required for efficient completion of the DNA damage response.

To investigate the mechanisms underlying SMYD3 function in this context, we focused on its interaction with ATM, the starter kinase that orchestrates HR-mediated repair [35]. Since SMYD3 is efficiently phosphorylated by active ATM [17], we characterized the SMYD3–ATM complex and its functional relevance in GC cells.

**Fig. 3:**
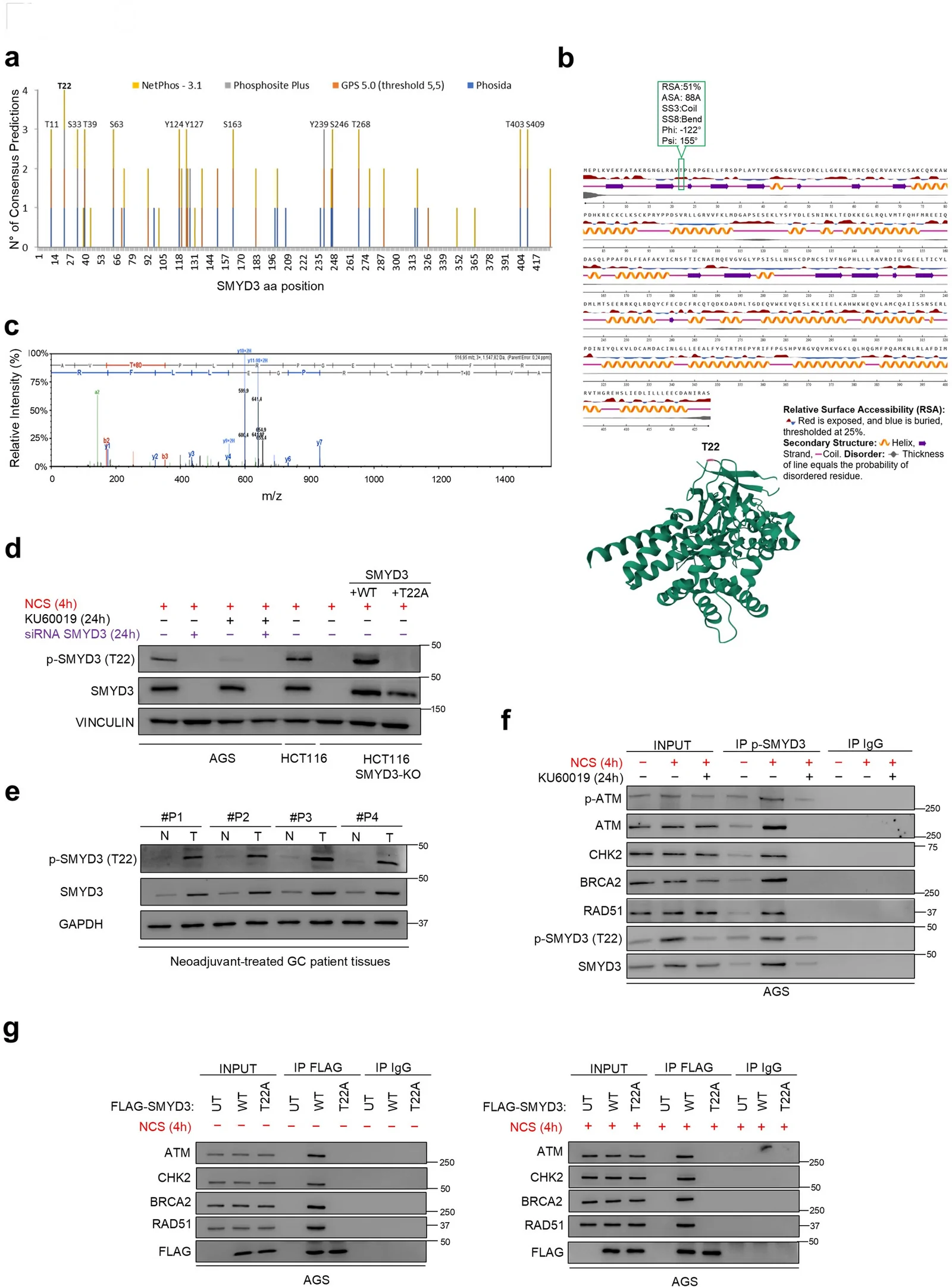
ATM-mediated phosphorylation of SMYD3 at T22 promotes HR and DDR. **a** Comparison of predicted phosphorylation sites along the SMYD3 protein sequence using multiple bioinformatic tools: NetPhos-3.1, Phosphosite Plus, GPS 5.0, and Phosida. Vertical bars indicate the predicted phosphorylation sites at specific amino acid positions. The x-axis shows the amino acid position along the protein sequence, while the y-axis represents the number or frequency of predicted phosphorylation events. Overlapping bars highlight regions with higher consensus among the different algorithms. **b** The relative surface accessibility of T22 was predicted *in silico* using NetSurfP-3.0. Exposed residues are shown in red, while buried residues are in blue, with secondary structure (helices in orange, coils in pink) and disordered regions (grey, line thickness proportional to disorder probability) indicated. **c** MS/MS spectrum of phospho-SMYD3 peptide generated by double trypsin digestion. **d** Immunoblot of SMYD3 T22 phosphorylation in AGS cells transfected with control or SMYD3-specific siRNAs (5 nM, 24 h), treated or not with KU60019 (5 µM, 24 h), and then exposed to NCS (1 nM, 4 h). HCT116 cells were used as controls: KO= knockout; SMYD3 WT= SMYD3-KO cells re-expressing exogenous SMYD3 WT; SMYD3 T22A= SMYD3-KO cells re-expressing SMYD3 T22A mutant. Vinculin was used as a loading control. **e** Immunoblot of the SMYD3 phosphorylation at T22 in protein extracts derived from GC patients’ tissues after neoadjuvant chemotherapy. GAPDH was used as a loading control. P = patient, N = normal, T = tumor. **f** Co-IP assay with anti-phospho-T22-SMYD3 antibody in nuclear fractions from AGS cells treated or not with NCS (1 nM, 4 h), with or without KU60019 pre-treatment (5 µM, 24 h). IgG was used as a negative control. **g** AGS cells were transiently transfected with FLAG-SMYD3-WT or SMYD3 mutant, FLAG-SMYD3-T22A, and treated or not with NCS (1 nM, 4 h). Immunoprecipitation was performed using an anti-FLAG antibody. IgG was used as a negative control. Data are representative of at least three independent experiments

To identify the SMYD3 phosphorylation sites targeted by ATM, we first performed an in silico meta-analysis using four tools (NetPhos-3.1, Phosphosite Plus, GPS 5.0, Phosida), which identified threonine 22 (T22) as the best candidate phosphorylation site (Fig. 3a). T22 is a surface-exposed residue in the SMYD3 protein structure, as shown by in silico analysis predicting the relative surface accessibility (RSA = 51%; threshold at 25%) of T22 (Fig. 3b, upper panel), as confirmed by its topological location in the SMYD3 three-dimensional structure (Fig. 3b, lower panel). Subsequently, to validate ATM-mediated phosphorylation of SMYD3-T22, we performed a mass spectrometry (MS) analysis of the recombinant SMYD3 protein after in vitro phosphorylation by ATM [17]. MS analysis revealed that phospho-SMYD3 was correctly identified with good coverage (64%), and the comparative peptide analysis between SMYD3 WT and phospho-SMYD3 allowed us to identify T22 as a residue phosphorylated exclusively in the phospho-SMYD3 sample at the level of peptide (20–32) AVTPLRPGELLFR (Fig. 3c).

Then, to evaluate SMYD3 phosphorylation in GC cells, we generated a custom antibody specific for SMYD3 phosphorylated at T22 (hereafter referred to as phospho-T22-SMYD3). To validate the specificity of this custom antibody, protein extracts were treated with recombinant Shrimp Alkaline Phosphatase (rSAP) or prepared in the absence of phosphatase inhibitors (PI) to promote dephosphorylation. In both conditions, the phospho-T22-SMYD3 signal was markedly reduced, confirming that the antibody specifically recognizes the phosphorylated T22 epitope rather than the non-phosphorylated threonine residue (Supplementary Fig. 2f). Having validated the specificity of the phospho-T22-SMYD3 antibody, we next investigated whether ATM induces SMYD3 T22 phosphorylation following DNA damage in GC cells. In AGS cells, immunoblotting confirmed that T22 phosphorylation is induced by ATM upon NCS treatment and abolished by ATM inhibition and/or SMYD3 silencing, demonstrating that this modification is ATM-dependent and triggered by DNA damage (Fig. 3d). Moreover, we used an independent genetic model based on HCT116 cells, comprising SMYD3 WT and CRISPR–Cas9-generated SMYD3 knockout (KO) cells. In HCT116-SMYD3-KO cells, SMYD3 expression was transiently restored by transfection with constructs encoding either SMYD3 WT or the phospho-deficient SMYD3-T22A mutant (Fig. 3d). Importantly, these findings were further corroborated through the analysis of protein extracts from GC patients’ tumor tissues treated with neoadjuvant chemotherapy. Compared with the corresponding normal tissues, tumoral samples exhibited elevated SMYD3 protein expression with a significant signal of T22-phosphorylation (Fig. 3e). Since chemotherapy induces DSBs and thereby activates ATM [36], these findings validated that SMYD3 phosphorylation occurs in vivo and suggested its functional relevance in the DDR within the tumor tissues.

Finally, to clarify the functional role of SMYD3 phosphorylation, we performed Co-IP assays using the phospho-T22-SMYD3 antibody in nuclear fractions from AGS cells after NCS treatment (Fig. 3f). These experiments showed that phospho-T22-SMYD3 interacts with key HR proteins (phospho-ATM, ATM, CHK2, BRCA2, and RAD51) and that ATM inhibition obtained with the KU60019 inhibitor reduces SMYD3 phosphorylation signal and the HR complex formation. This indicates that ATM activity is required for DNA damage-induced phosphorylation of SMYD3 and its association with HR protein complexes. To determine whether ATM-dependent phosphorylation of SMYD3 at T22 is critical for HR complex assembly, AGS cells were transiently transfected with FLAG-SMYD3-WT or the phospho-deficient FLAG-SMYD3-T22A mutant and exposed to NCS. FLAG Co-IP assays revealed that, following DNA damage, FLAG-SMYD3-WT efficiently associated with key HR proteins, including ATM, CHK2, BRCA2, and RAD51 (Fig. 3g). In contrast, the phospho-deficient T22A mutant failed to associate with these proteins, demonstrating that ATM-dependent phosphorylation of SMYD3 at T22 is required for the assembly of HR repair complexes in response to DNA damage (Fig. 3g). To investigate the functional consequences of T22 phosphorylation, KATOIII cells, which express low endogenous levels of SMYD3, were transiently transfected with FLAG-SMYD3-WT or the phospho-deficient FLAG-SMYD3-T22A mutant and exposed to NCS. Cell proliferation was evaluated by the WST assay 72 h after DNA damage induction. Compared with cells expressing SMYD3-WT, cells expressing the T22A mutant exhibited significantly reduced proliferation (Supplementary Fig. 2g). Consistent with these findings, immunoblot analysis showed increased levels of cleaved PARP in T22A-expressing cells, indicating enhanced apoptosis (Supplementary Fig. 2h). Together, these results demonstrate that ATM-dependent phosphorylation of SMYD3 at T22 is essential not only for HR complex assembly but also for promoting cell survival following DNA damage.

### Targeting SMYD3 enhances the PARP inhibitors’ response in GC cell lines

**Fig. 4:**
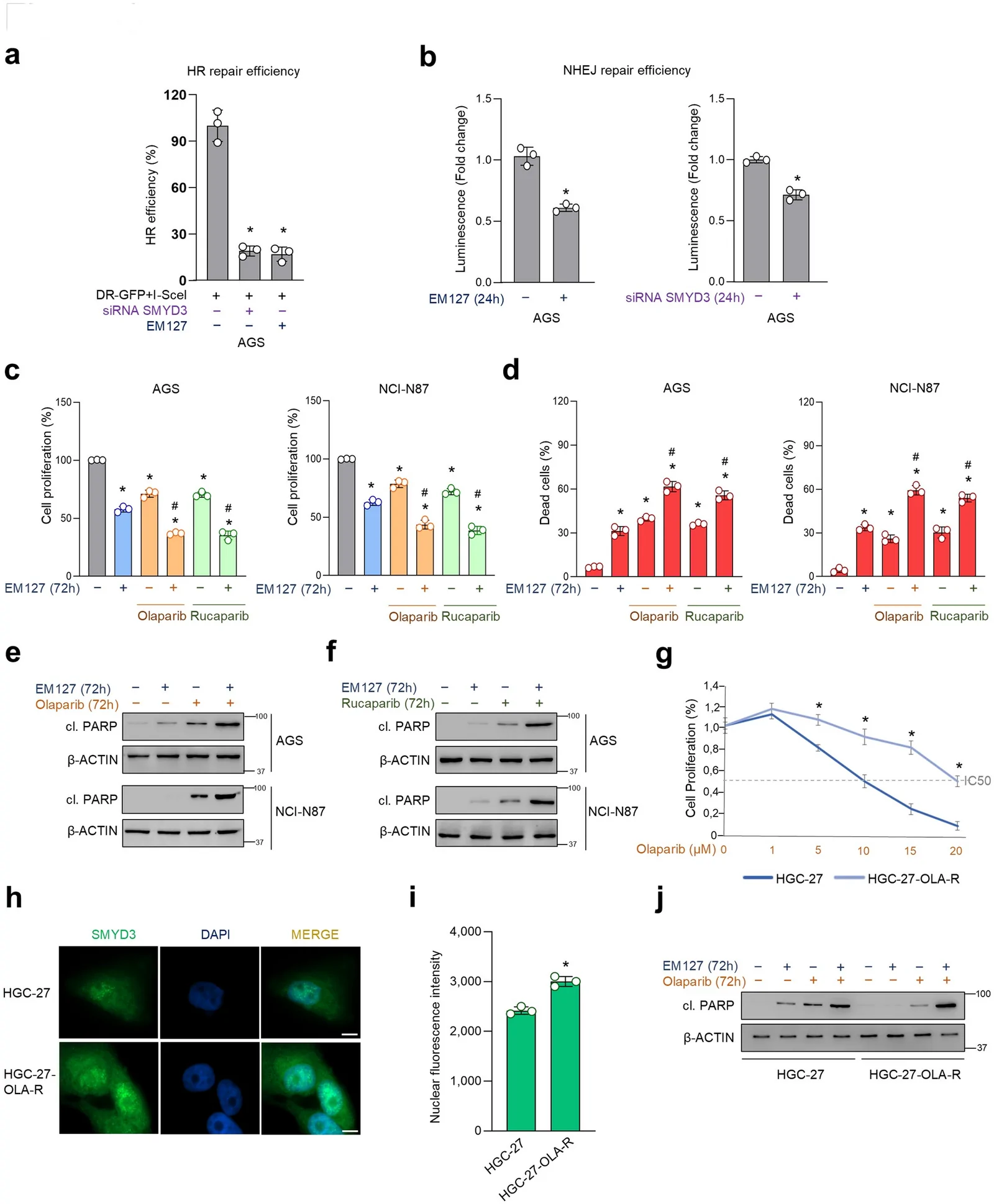
SMYD3 inhibition sensitizes GC cells to PARPis and overcomes acquired olaparib resistance.** a** Effect of SMYD3 knockdown or pharmacological inhibition on DR-GFP HR reporter activity in AGS cells. HR activity was quantified as GFP-positive cells 72 h after co-transfection of DR-GFP and pCBASce. **b** AGS cells were pre-treated or not with EM127 (5 µM) or transfected with SMYD3 siRNA (5 nM). Cells were transfected with the linearized or uncut pGL3 promoter vector. After 24 h, NHEJ activity was measured by normalizing the luciferase activity of the linearized pGL3 signal to the uncut pGL3 control signal. **c** Cell proliferation (CellTiter 96 AQueous assay) in AGS and NCI-N87 GC cells treated or not with EM127 (5 µM) alone or in combination with olaparib (10 µM) or rucaparib (5 µM) for 72 h. **d** Cell death (trypan blue staining) in AGS and NCI-N87 cells under the same treatment conditions as in (**c**). **e-f** Immunoblot analysis of cl. PARP in AGS and NCI-N87 cells treated or not with EM127 (5 µM), alone or in combination with olaparib (10 µM) (**e**) or rucaparib (5 µM) (**f**) for 72 h. β-ACTIN was used as a loading control. **g** Quantification of cell proliferation by CellTiter 96 AQueous Assay in HGC-27 and HGC-27-OLA-R cells in response to 72 h of treatment with different doses of olaparib (0–20 µM). **h-i** Immunofluorescence analysis and quantification of nuclear SMYD3 expression (green) in HGC-27 and HGC-27-OLA-R cells. Nuclei were counterstained with DAPI (blue). Scale bar: 5 μm. **j** Immunoblot analysis of cl. PARP in HGC-27 and HGC-27-OLA-R cells treated or not with EM127 (5 µM), and/or olaparib (10 µM) for 72 h. β-ACTIN was used as a control. cl. PARP = cleaved PARP. Data are representative of at least three independent experiments. Significance was determined by Student’s t-test (*p < 0.05 treated vs. untreated; #*p* < 0.05 combined vs. single treatments)

We first assessed the impact of SMYD3 depletion and pharmacological inhibition on HR repair in the AGS GC cell line using the DR-GFP reporter assay. Efficient SMYD3 knockdown following siRNA transfection was confirmed by Western blot analysis (Supplementary Fig. 2i). Both siRNA-mediated SMYD3 knockdown and EM127 pretreatment caused a decrease in HR efficiency in AGS cells (Fig. 4a), confirming that SMYD3 regulates HR activity also in GC cells. Beyond its role in HR, SMYD3 also appears to influence the NHEJ pathway in CRC cell lines [14]. We therefore investigated whether SMYD3 similarly affects NHEJ repair in GC cells. Using an NHEJ reporter assay in AGS cells (Fig. 4b), we observed that both SMYD3 silencing and pharmacological inhibition partially impaired NHEJ repair efficiency, consistent with previous studies [14, 37], further supporting a role for SMYD3 in modulating this repair pathway. Overall, our molecular analyses indicate that SMYD3 inhibition markedly suppresses HR while exerting a more limited effect on NHEJ. Based on these observations, we hypothesized that SMYD3 inhibition could enhance the sensitivity of cancer cells to PARPis, which impair DNA repair processes and preferentially induce cell death in tumors with defective HR. Therefore, the combined inhibition of SMYD3 and PARP is expected to further compromise DNA repair, promote the accumulation of unresolved DNA damage, and enhance cancer cell death through a synthetic lethality mechanism. This rationale provides a strong mechanistic basis for exploring SMYD3 inhibition as a potential strategy to improve the therapeutic efficacy of PARPis. Then, we based our approach on previous studies in CRC and breast cancer cells, treating cells with the SMYD3 inhibitor BCI-121, alone or in combination with the PARPi olaparib, for 72 h, and assessing cell death in single and dual treatment conditions [17]. In the present study, we extended this approach to GC cell lines and confirmed similar effects: combined inhibition of SMYD3 and PARP induced apoptosis in high-SMYD3 cells, AGS and NCI-N87, as indicated by the apoptotic marker cleaved PARP, whereas low-SMYD3 KATOIII cells showed no significant response (Supplementary Fig. 3a-b). The HGC-27 cell line, known to be PARPi-sensitive [25, 26], was included as a control. Consistently, trypan blue assays revealed an increase in cell death in high-SMYD3 cells following combined treatment (Supplementary Fig. 3c).

We next evaluated the effects of combining PARP inhibition with the SMYD3 inhibitor EM127, which irreversibly and covalently inhibits SMYD3 with higher potency and selectivity than BCI-121. Dose–response analyses in AGS and NCI-N87 cells showed that EM127 inhibited cell viability at markedly lower concentrations than BCI-121 (Supplementary Fig. 3d–e), confirming EM127 as the preferred compound for functional assays and combinatorial therapeutic approaches.

We assessed the combined inhibition of SMYD3 and PARP in AGS and NCI-N87 cells. Olaparib and another clinically relevant PARPi currently in GC trials [6], rucaparib, were tested as monotherapy or in combination with EM127. Cellular assays for proliferation and cell death (Fig. 4c-d) revealed a pronounced reduction in cell proliferation and increased cell death in combined treatments compared with monotherapies, as confirmed by PARP cleavage (Fig. 4e-f, Supplementary Fig. 3f-g). Importantly, rucaparib reproduced the effects of olaparib, supporting a class-wide effect of PARP inhibition in combination with SMYD3 targeting and underscoring the clinical relevance of this therapeutic strategy (Fig. 4f, Supplementary Fig. 3g).

Given that acquired resistance limits the clinical efficacy of PARPis, we generated an olaparib-resistant HGC-27 cell line (HGC-27-OLA-R) by chronic exposure to increasing inhibitor concentrations. Resistant cells showed reduced sensitivity to olaparib in proliferation assays (Fig. 4g). Immunofluorescence analysis revealed significantly increased nuclear SMYD3 expression in resistant cells compared with the parental line, suggesting its role in mediating PARPi resistance (Fig. 4h-i). Accordingly, pharmacological inhibition of SMYD3 with EM127 restored olaparib sensitivity in HGC-27-OLA-R cells, as indicated by increased PARP cleavage (Fig. 4j, Supplementary Fig. 3h). Importantly, consistent with the pharmacological inhibition results, SMYD3 genetic silencing markedly enhanced olaparib-induced PARP cleavage (Supplementary Fig. 3i), confirming the specificity of SMYD3 targeting in restoring PARPis sensitivity, and demonstrating that SMYD3 targeting can overcome acquired resistance in this GC model.

### Tumorsphere and patient-derived organoid models reveal the therapeutic potential of SMYD3 inhibition in GC

**Fig. 5:**
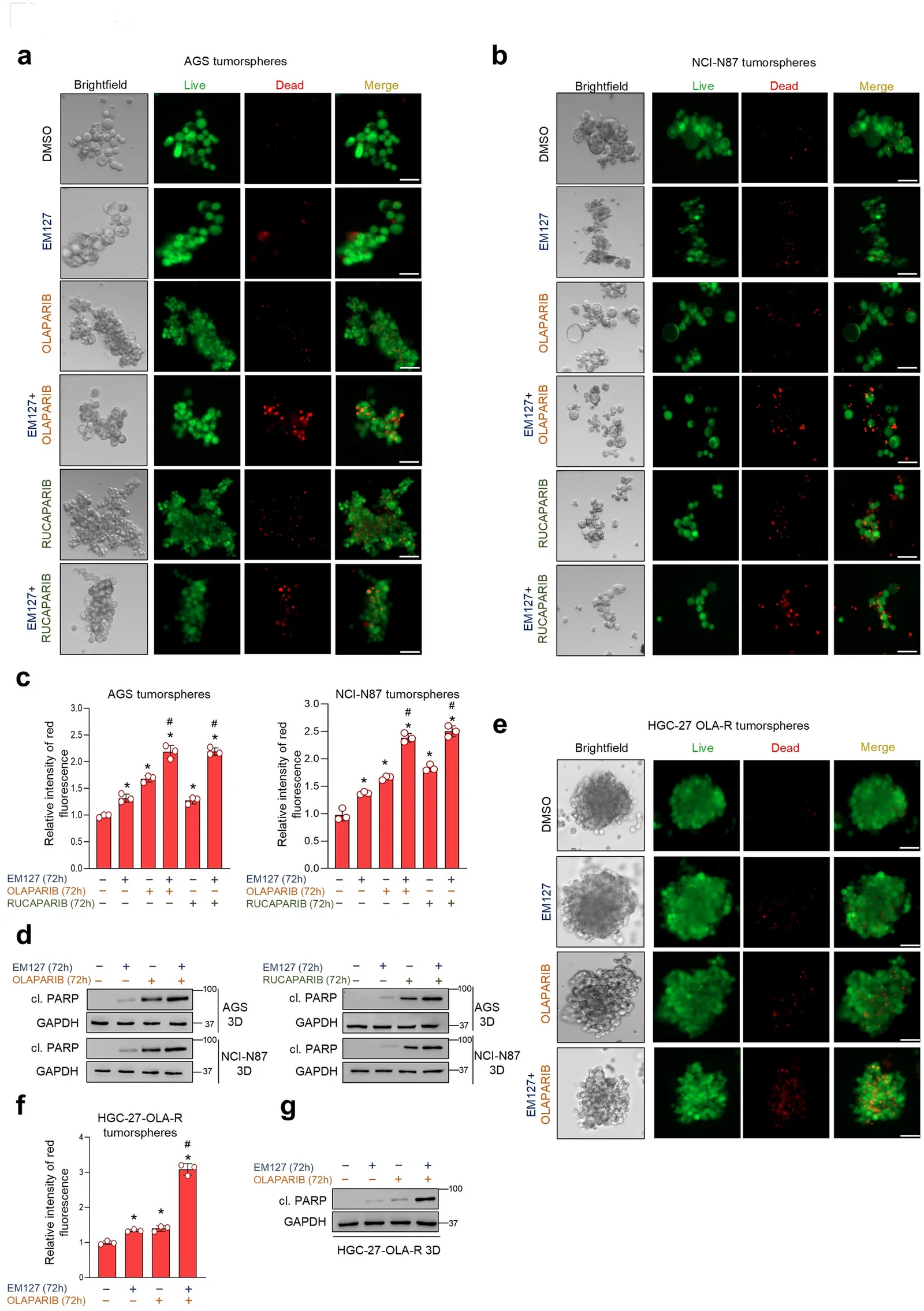
SMYD3 inhibition enhances PARPi–induced cytotoxicity in tumorspheres.** a-b** Live/dead staining of AGS **(a)** and NCI-N87 **(b)** cells grown as tumorspheres, treated or not with EM127 (5 µM), alone or in combination with olaparib (10 µM) or rucaparib (5 µM) for 72 h. Green: live cells; red: dead cells. Scale bar: 100 μm. **c** The graph shows the mean intensity of dead cell staining (red fluorescence) in AGS and NCI-N87 tumorspheres. **d** Immunoblot analysis of cl. PARP in AGS and NCI-N87 tumorspheres, treated as in **(a**,** b)**. GAPDH was used as a loading control. **e** Live/dead staining of HGC-27-OLA-R tumorspheres, treated or not with EM127 (5 µM), and/or with olaparib (10 µM) for 72 h. Green: live cells; red: dead cells. Scale bar: 100 μm. **f** The graph shows the mean intensity of dead cell staining (red fluorescence) in HGC-27-OLA-R tumorspheres. **g** Immunoblot analysis of cl. PARP in HGC-27-OLA-R tumorspheres, treated as in **(e)**. GAPDH was used as a loading control. cl. PARP = cleaved PARP, DMSO = dimethyl sulfoxide. Data are representative of at least three independent experiments, and significance was determined by Student’s t-test (*p < 0.05 treated vs. untreated; #*p* < 0.05 combined treatment vs. single treatments)

To extend these findings to an experimental model that better recapitulates tumor complexity, we generated GC cell-derived tumorspheres and characterized them for stemness-associated markers (i.e., KLF4, OCT4) and SMYD3 expression. Compared with monolayer cultures, tumorspheres displayed increased levels of KLF4 and OCT4, together with elevated SMYD3 expression, supporting a potential role for SMYD3 in tumorsphere-forming ability (Supplementary Fig. 4a-b). We next evaluated the therapeutic relevance of targeting SMYD3 in this context by treating tumorspheres with the SMYD3 inhibitor EM127 and the PARPis olaparib or rucaparib, alone or in combination. Live/dead staining revealed that combined inhibition led to a marked reduction in tumorsphere viability and an increase in cell death in both 3D GC models, compared with single treatments (Fig. 5a–c). These findings were further corroborated by increased levels of cleaved PARP following combination treatment (Fig. 5d; Supplementary Fig. 4c), indicating a strong induction of apoptosis. Collectively, these findings indicate that SMYD3 inhibition synergizes with PARPis to induce synthetic lethality in GC cells, and this vulnerability is retained in tumorsphere models. In HGC-27-OLA-R–derived tumorspheres, SMYD3 inhibition restored olaparib sensitivity and significantly reduced viability when combined with PARP inhibition (Fig. 5e–f), supported by increased PARP cleavage (Fig. 5g, Supplementary Fig. 4d). Together, these results show that SMYD3 inhibition re-sensitizes PARPi-resistant GC cells in both 2D and 3D models.

**Fig. 6:**
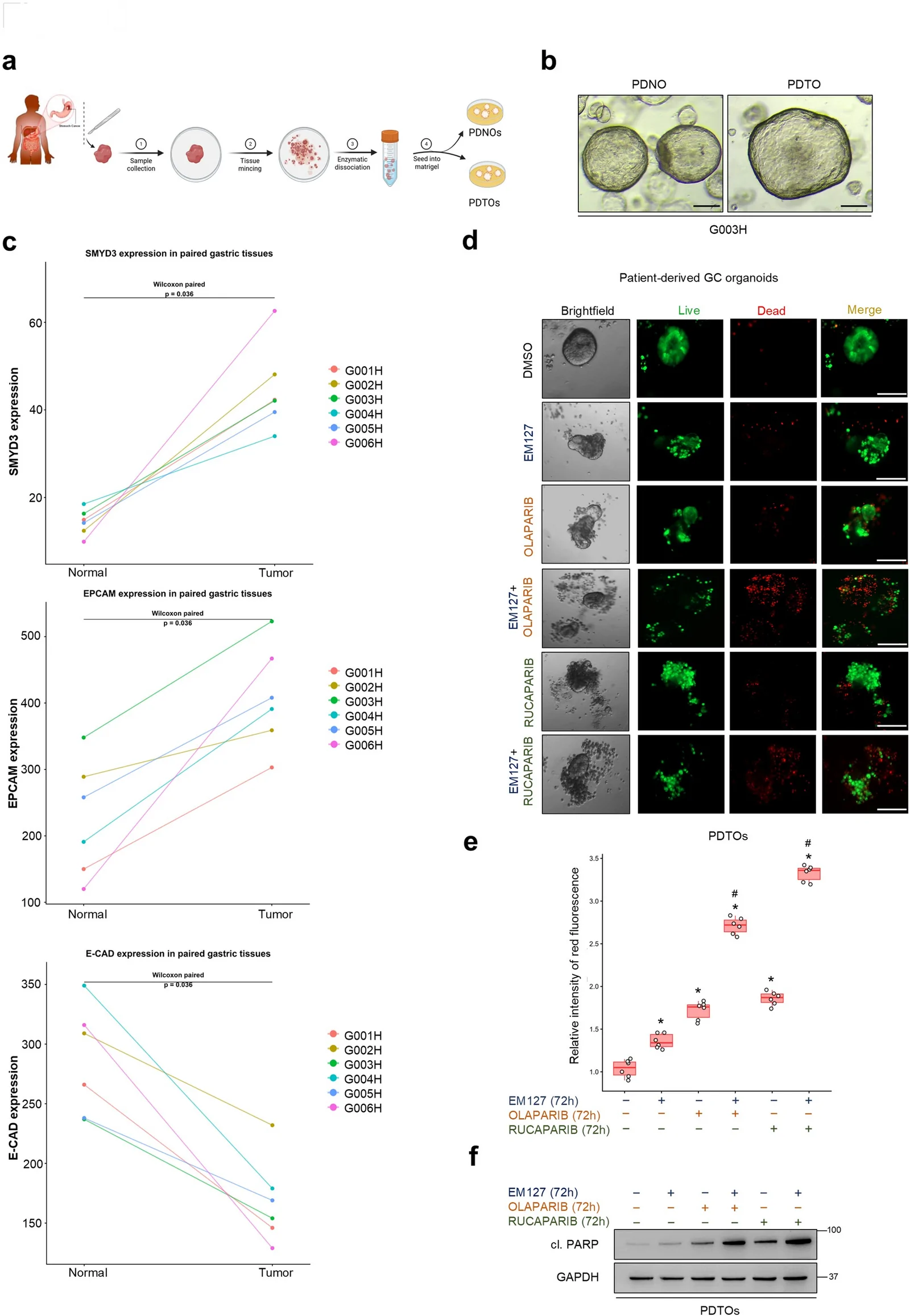
SMYD3 inhibition enhances PARPi–induced cytotoxicity in PDTOs.** a** Schematic representation of the experimental processing of GC organoids created in BioRender. **b** Representative brightfield images of normal and tumor PDOs, grown from single cells in media supplemented with 50% Matrigel. Scale bar: 200 μm. **c** ddPCR analysis revealed the GC markers EpCAM and CDH1, as well as SMYD3, in PDNOs and PDTOs. Statistical analysis was performed using a paired Wilcoxon test. **d** Live/dead staining of PDTOs, treated or not with EM127 (5 µM), and treated or not with olaparib (10 µM), or rucaparib (5 µM), for 72 h. Green: live cells; red: dead cells. Scale bar: 200 μm. **e** The graph shows the relative intensity of dead cell staining (red fluorescence) in PDTOs. **f** Immunoblot analysis of cl. PARP in PDTOs, treated or not with EM127 (5 µM), alone or in combination with olaparib (10 µM), or rucaparib (5 µM) for 72 h. GAPDH was used as a loading control. DMSO = dimethyl sulfoxide. cl. PARP = cleaved PARP. Data are representative of at least three independent experiments. Significance was determined by Student’s t-test (*p < 0.05 treated vs. untreated; #*p* < 0.05 combined treatment vs. single treatments)

Finally, to further validate the translational relevance of this therapeutic strategy, we collected normal and pathological tissues from GC patients undergoing surgical resection at the IRCCS “S. De Bellis” – Research Hospital in Castellana Grotte (Bari, Italy). These tissue samples were processed for droplet digital PCR (ddPCR) analysis to determine SMYD3 expression levels and for the generation of matched PDOs, including normal (PDNOs) and tumor (PDTOs) organoids (Fig. 6a–b). ddPCR analysis identified a subset of tumors with significantly higher SMYD3 expression than their matched normal tissues (Supplementary Fig. 4e); specifically, increased SMYD3 expression was observed in approximately 50% of the tumors analyzed. Based on this molecular stratification, only PDNOs and PDTOs derived from the SMYD3-high patient cohort (G001H, G002H, G003H, G004H, G005H, and G006H) were selected for further characterization. The expression of the GC markers EpCAM and CDH1, together with SMYD3, was then assessed by ddPCR in PDNOs and PDTOs (Fig. 6c). This analysis revealed distinct molecular profiles between PDNOs and PDTOs: EpCAM was enriched in PDTOs, consistent with its epithelial and tumor-associated role in GC [38], whereas CDH1 was reduced in PDTOs, reflecting loss of epithelial integrity and increased invasiveness [39]. Importantly, elevated SMYD3 expression was maintained in PDTOs compared with matched PDNOs (Fig. 6c), confirming the tumor-specific nature of these organoids and providing a relevant model to study the therapeutic implications of SMYD3 targeting.

Then, PDTOs were treated with EM127, olaparib, or rucaparib, alone or in combination. Consistent with our data from 2D and 3D GC models, live/dead staining showed that dual inhibition of SMYD3 and PARP significantly reduced PDTO viability compared with single-inhibitor treatments, resulting in marked cell death (Fig. 6d-e). These data were further supported by increased cleaved PARP levels (Fig. 6f, Supplementary Fig. 4f).

Collectively, these results confirmed that combined inhibition of SMYD3 and PARP could represent a valid strategy to induce cancer cell death by a synthetic lethality approach, and this effect in PDTOs highlights the translational potential of SMYD3 and PARP co-inhibition as a promising precision strategy for GC.

## Discussion

In this study, we extend our previous findings implicating SMYD3 in the HR pathway by defining the molecular mechanisms through which it regulates DNA DSB repair. Specifically, we show that ATM-dependent phosphorylation of SMYD3 promotes chromatin remodeling at damaged sites, facilitating HR factors recruitment and influencing repair pathway choice. Consistent with this mechanism, SMYD3 inhibition disrupts the chromatin landscape at DSBs, impairing efficient activation of repair pathways. SMYD3 is overexpressed in GC and associates with key HR proteins, further supporting its involvement in the DDR. Our findings provide mechanistic insight into this previously reported role by defining how SMYD3 regulates DSB repair. Mechanistically, we showed that SMYD3 is recruited to HR-prone DSBs, where it shapes histone modifications to create a chromatin environment permissive for HR protein recruitment. Concurrently, it limits 53BP1 accumulation, acting as an upstream regulator of repair pathway choice.

Histone-modifying enzymes are widely recognized as key DDR regulators and have emerged as promising therapeutic targets [40]. In particular, histone deacetylase (HDAC) inhibitors have demonstrated significant therapeutic potential, with several pan-HDAC inhibitors already approved for hematological malignancies, while others are under investigation for solid tumors [41]. In GC, the HDAC inhibitor vorinostat induces apoptosis and autophagy [42], whereas panobinostat is currently being evaluated in phase II clinical trials [43], supporting its use in combination strategies [43, 44]. Next-generation inhibitors, such as trichostatin A, aim to improve specificity while maintaining antitumor activity [45]. Beyond HDACs, histone methyltransferases represent another important class of targetable epigenetic regulators. Specifically, inhibitors of DOT1L and EZH2 are emerging as promising therapeutic strategies in GC [46]. DOT1L, which has been implicated in tumor progression and metastasis, can be inhibited by EPZ-5676, a selective compound currently evaluated in clinical trials for hematologic malignancies [47–49]. Similarly, natural compounds such as hesperetin can modulate epigenetic pathways, and hesperetin has been shown to reduce DOT1L expression and H3K79 methylation [46]. EZH2 is one of the most studied histone methyltransferases due to its frequent overexpression in cancer and its role in tumor progression [50, 51]. Although several EZH2 inhibitors are under investigation, tazemetostat remains the only FDA-approved drug for metastatic or locally advanced epithelioid sarcoma and follicular lymphoma [52, 53]. It acts as a competitive EZH2 inhibitor, reducing H3K27 trimethylation and reactivating silenced tumor suppressor genes [53]. Its therapeutic potential in solid tumors is currently being evaluated in clinical trials [51, 54]. In GC, EZH2 overexpression is associated with poor prognosis, tumor progression, metastasis, and therapeutic resistance [55]. GC is frequently diagnosed at advanced stages due to the late onset of symptoms, and epigenetic alterations may arise before the tissue becomes histologically malignant [56]. This makes epigenetic therapies attractive not only for the treatment of GC, but also for early detection, disease staging, and prognostic prediction [46]. Since epigenetic modifications are reversible, they represent attractive therapeutic targets that can be exploited in combination with conventional or targeted therapies to improve treatment efficacy in solid tumors [57]. In this context, our findings reveal the molecular basis through which SMYD3 coordinates chromatin dynamics and repair factor assembly at DSBs, uncovering a previously unrecognized chromatin vulnerability in GC. Consistent with this role, SMYD3 inhibition disrupts HR factor recruitment and impairs HR, while only moderately affecting NHEJ. Therapeutically, this suggests that targeting SMYD3 may induce a “BRCAness” state in HR-proficient tumors, thereby expanding PARPi applicability. In SMYD3-high GC models, combined SMYD3 and PARP inhibition abrogates both HR and NHEJ, leading to the accumulation of unrepaired DNA and increased apoptosis through a synthetic lethality mechanism. The translational relevance of this strategy is supported by tumorspheres and PDOs, which better recapitulate tumor physiology than monolayer cultures [58]. PDOs preserve key genetic and epigenetic features of primary tumors, providing a platform to evaluate targeted therapies, including patient-specific responses and biomarkers [59]. Notably, SMYD3-high PDTOs are highly sensitive to combined SMYD3 and PARP inhibition, supporting SMYD3 as a predictive biomarker for DDR-targeted therapies.

## Conclusion

In summary, our findings define a subset of GCs characterized by SMYD3 overexpression, limited actionable alterations, and mutual exclusivity with key DDR genes, highlighting SMYD3 as a potential therapeutic target. By extending the applicability of PARPis beyond BRCA-mutated settings, SMYD3 inhibition may broaden the treatment options for GC, providing a strong rationale for its clinical exploitation and paving the way toward novel therapeutic strategies.

## Supplementary Information

Supplementary Material 1: Supplementary Fig. 1. a, SMYD3 mRNA expression (log2[TPM + 1], DepMap Public 26Q1) across 41 GC cell lines, ranked from the highest to the lowest expressing. The heatmap on the left shows SMYD3 mRNA expression [log2(TPM + 1)]; the heatmap on the right shows the fold change of each cell line relative to the median of the SMYD3-low reference group (lowest-expressing quartile, n = 11; bracketed on the right), which includes KATOIII. The cell lines used in this study are outlined: AGS, HGC-27, and NCI-N87 (green) express 4.4-, 2.8-, and 1.6-fold higher SMYD3 mRNA than the reference, whereas KATO III (red, dashed) belongs to the SMYD3-low reference group. b, SMYD3 protein in the 14 gastric cell lines, ranked from the highest to the lowest expressing. The heatmap on the left shows SMYD3 protein level (mass-spectrometry log2 ratio); the heatmap on the right shows the fold-change of each cell line relative to the median of the SMYD3-low reference group (lowest-expressing quartile, n = 4; bracketed on the right), which includes KATOIII. AGS, HGC-27, and NCI-N87 (green outline) show 4.8-, 5.8-, and 1.6-fold higher SMYD3 protein than the reference. c, immunoblot analysis shows the SMYD3 protein levels in GC and CRC cell lines. GAPDH was used as a loading control. d, Table shows the mutational status of key HR-related genes and SMYD3 expression levels in GC cell lines. e, Co-IP analysis of HR proteins and SMYD3 in AGS and NCI-N87 cell lines using the indicated antibodies. Immunoprecipitation with IgG was used as a negative control. Data are representative of at least three independent experiments.

Supplementary Material 2: Supplementary Fig. 2. a, Immunofluorescence analysis of AGS cells transfected with the HA-tagged ER-AsiSI system, co-stained with DAPI (nucleus) after incubation with antibodies against the HA-tag (green) and γH2AX (red), before and after 4OHT treatment. Scale bar: 5 μm. b, The graph shows the percentage of γH2AX-positive nuclei in 4OHT–treated versus untreated ER-AsiSI-AGS cells. c, The table summarizes the AsiSI position, the associated gene regions, the closest genes, and their classification as DSB repair pathway prone to AsiSI sites analyzed, together with a non-cleaved control locus. d, ChIP analysis of 53BP1 binding at the DSB3 site in the indicated conditions. Chromatin was immunoprecipitated using an anti-53BP1 antibody, and enrichment at the DSB3 site was quantified by RT-qPCR. Quantification was performed using the % input method. e, Representative immunofluorescence images of AGS cells with NCS (1 nM) for 24 h in the presence or absence of EM127 (5 µM) or after transfection with control siRNA or SMYD3 siRNA (5 nM). Cells were immunostained for γH2AX (green), and nuclei were counterstained with DAPI (blue). Scale bar: 5 μm. f, Protein extracts were treated with recombinant shrimp alkaline phosphatase (rSAP) and/or prepared in the absence of phosphatase inhibitors (PI) to promote dephosphorylation following NCS treatment (1 nM, 4 h). VINCULIN was used as a control. g, KATOIII cells were transfected or not with wild-type SMYD3 (SMYD3-WT) or the SMYD3 mutant (SMYD3-T22A) and treated with NCS for 24, 48, and 72 h. Cell proliferation was determined by WST assay at the indicated time points. Data are rapresentative of at least three independent experiments. h, cell lysates of KATOIII cells transfected or not with SMYD3-WT or the SMYD3-T22A mutant and treated with NCS (1 nM) for 72 h were analyzed by immunoblot analysis. FLAG antibody and β-Actin were used as controls. cl. PARP = cleaved PARP; statistical analysis was performed by Student’s test (*p < 0.05, 4OHT-treated vs. untreated). i, Validation of SMYD3 knockdown in AGS cells by Western blot analysis following siRNA transfection. VINCULIN was used as a loading control.

Supplementary Material 3: Supplementary Fig. 3. a, Immunoblot analysis of cl. PARP levels in GC cell lines treated or not with BCI-121 (100 µM), and/or olaparib (10 µM), for 72 h. GAPDH was used as a loading control. b, Densitometric quantification of the immunoblot of cl. PARP, normalized to GAPDH, in the indicated GC cell lines. c, GC cell lines were treated or not with BCI-121 (100 µM) and/or olaparib (10 µM) for 72 h. Cell death in GC cell lines was quantified by trypan blue staining. d-e, Dose–response analysis of the effects of the SMYD3 inhibitors BCI-121 (d) and EM127 (e) in AGS and NCI-N87 GC cell lines. GC cells were treated with various concentrations of BCI-121 (0, 10, 30, 60, 100 µM) or EM127 (0, 0.5, 1, 2.5, 5 µM) for 72 h. f–h, Densitometric quantification of cl. PARP immunoblots shown in Fig. 4e (f), Fig. 4f (g), and Fig. 4j (h), normalized to β-ACTIN. i, Immunoblot analysis of cl. PARP in HGC-27 parental and HGC-27-OLA-R cell lines transfected with control siRNA or SMYD3 siRNA, treated or not with olaparib (10 µM) for 72 h. β-ACTIN was used as a loading control. Data are representative of at least three independent experiments. Significance was calculated using Student’s t-test; *p < 0.05 was considered statistically significant. cl. PARP = cleaved PARP. Data are representative of at least three independent experiments.

Supplementary Material 4: Supplementary Fig. 4. a, Immunoblot analysis of cancer stem cell markers KLF4 and OCT4 and SMYD3 expression levels in 2D vs. 3D GC models. GAPDH was used as a loading control. b, Densitometric quantification of the immunoblot of KLF4, OCT4, and SMYD3 normalized to GAPDH in the indicated GC cell lines. c, Densitometric quantification of the immunoblot of cl. PARP shown in Fig. 5d, normalized to GAPDH in AGS and NCI-N87 GC cell lines. d, Densitometric quantification of the immunoblot of cl. PARP shown in Fig. 5g, normalized to GAPDH in HGC-27-OLA-R GC cells. e, ddPCR analysis revealed the expression levels of SMYD3 in normal and pathological tissues from 12 GC patients. P = patient. f, Densitometric quantification of cl. PARP immunoblot shown in Fig. 6f, normalized to GAPDH in PDTOs. cl. PARP = cleaved PARP. Data are representative of at least three independent experiments. Significance was determined by Student’s t-test (*p < 0.05 was considered statistically significant).

Supplementary Material 5.

## Data Availability

The original data presented in this study are openly available in FigShare at https://doi.org/10.6084/m9.figshare.33425635 and https://doi.org/10.6084/m9.figshare.33425479.

## Abbreviations

ChIP: Chromatin immunoprecipitation
cl. PARP: cleaved PARP
Co-IP: Co-immunoprecipitation
COSMIC: Catalogue of Somatic Mutations in Cancer
CRC: Colorectal cancer
CTR: Control
ddPCR: Droplet digital PCR
DDR: DNA damage response
DSB(s): double-strand break(s)
DTT: Dithiothreitol
ER: Estrogen receptor
FBS: Fetal bovine serum
GC: Gastric cancer
H4K20me2: Histone H4 lysine 20 dimethylation
H3K9me3: Histone H3 lysine 9 trimethylation
H4K16ac: Histone H4 lysine 16 acetylation
H3K79me2: Histone H3 lysine 79 dimethylation
HDAC: Histone deacetylase
HGC-27-OLA-R: olaparib-resistant HGC-27 cell line
HR: Homologous recombination
KO: Knockout
IAA: Iodoacetamide
MS: Mass spectrometry
MSI-H: High microsatellite instability
NCS: Neocarzinostatin
NEEA: Non-essential amino acids
NHEJ: Non-homologous end joining
PARP: Poly(ADP-ribose) polymerase
PARPi(s): PARP inhibitor(s)
PDNO(s): Patient-derived normal organoid(s)
PDO(s): Patient-derived organoid(s)
PDTO(s): Patient-derived tumor organoid(s)
PI: Phosphatase inhibitor
RSA: Relative surface accessibility
rSAP: Recombinant Shrimp Alkaline Phosphatase
STAD: Stomach adenocarcinoma
TCGA: The Cancer Genome Atlas
TMB-H: High tumor mutation burden
T22: Threonine 22
WT: Wild-type
4OHT: 4-hydroxytamoxifen
