# Supplementary material for: Targeting the DNA-repair chromatin-modifier protein SMYD3 as a novel epigenetics-based therapy for gastric cancer
Source: J Exp Clin Cancer Res. 2026 Sep 12;45:202. doi: 10.1186/s13046-026-03823-2 (PMC13602472; doi:10.1186/s13046-026-03823-2)

Figure 1f

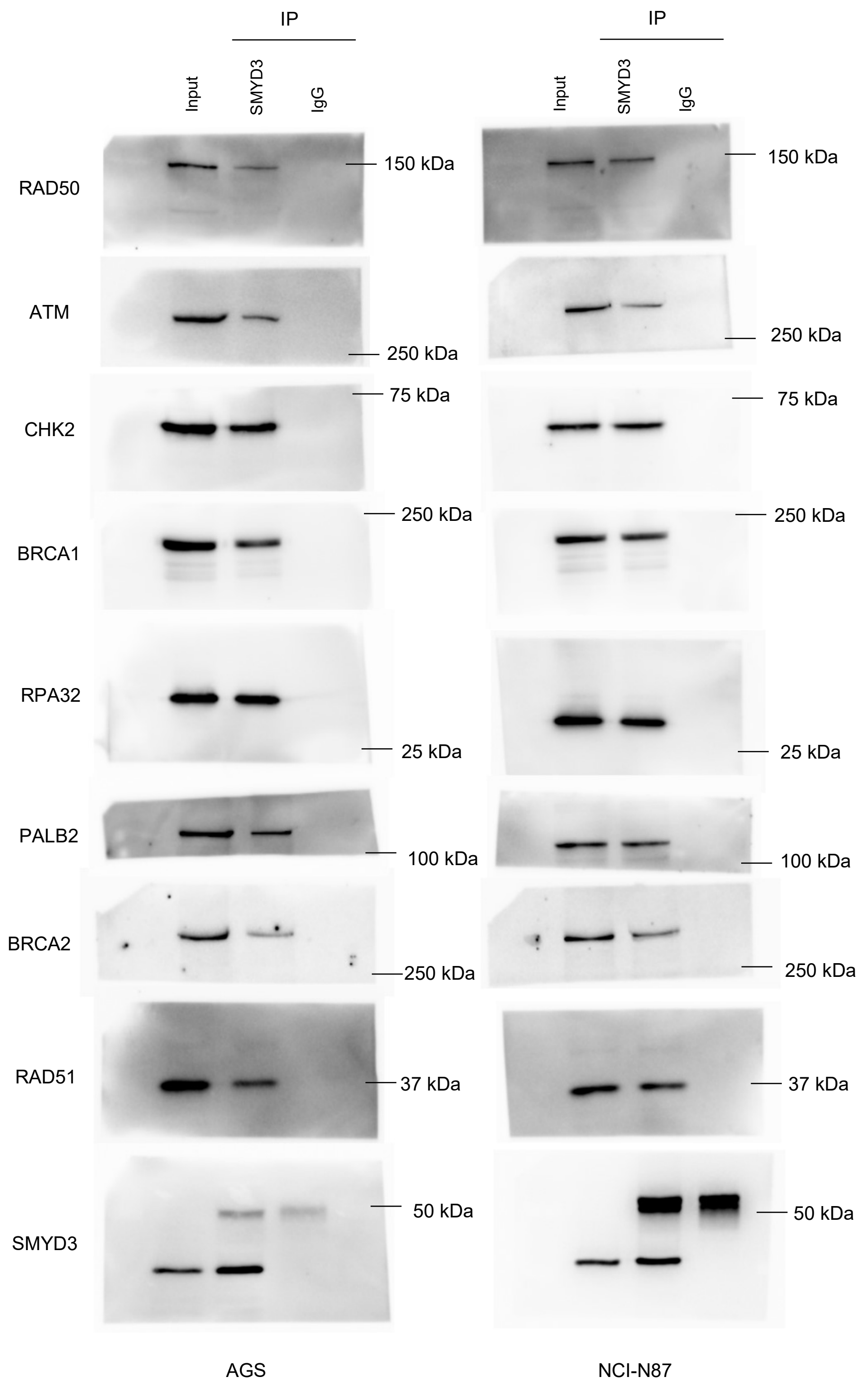

IP
Input
SMYD3
IgG
IP
Input
SMYD3
IgG
RAD50
150 kDa
150 kDa
ATM
250 kDa
250 kDa
CHK2
75 kDa
75 kDa
BRCA1
250 kDa
250 kDa
RPA32
25 kDa
25 kDa
PALB2
100 kDa
100 kDa
BRCA2
250 kDa
250 kDa
RAD51
37 kDa
37 kDa
SMYD3
50 kDa
50 kDa
AGS
NCI-N87

Figure 3d

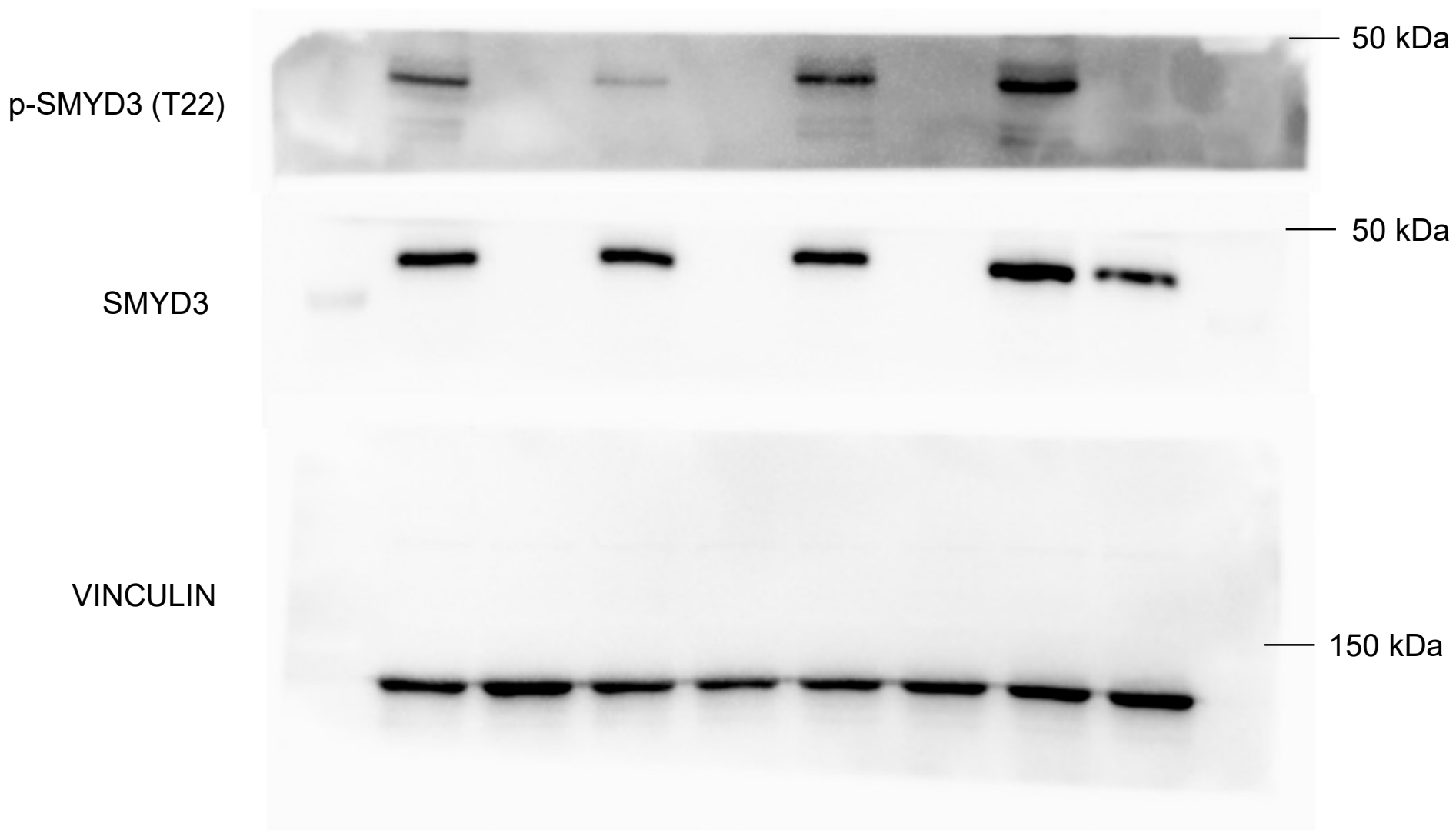

Figure 3e

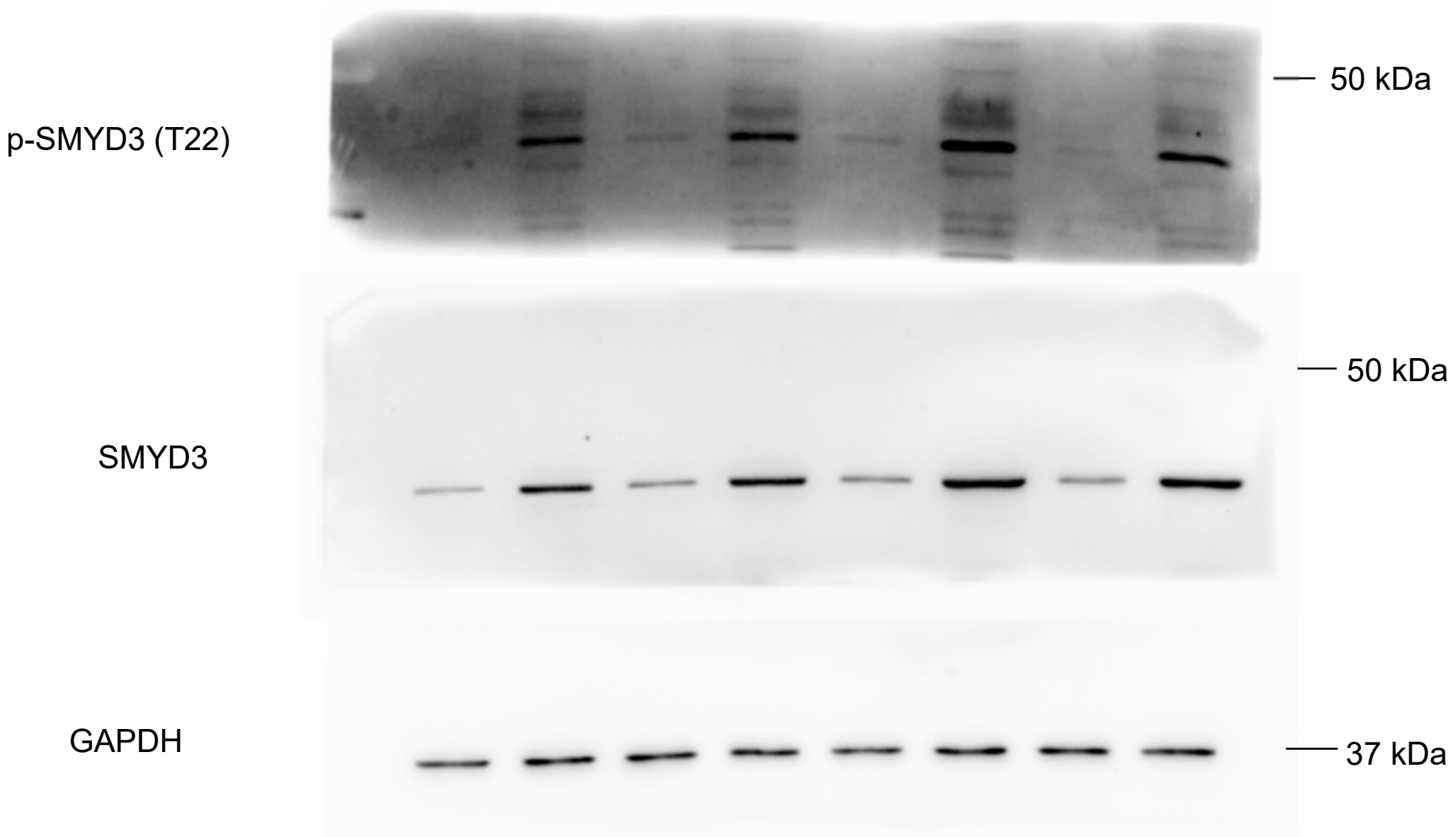

Figure 3f

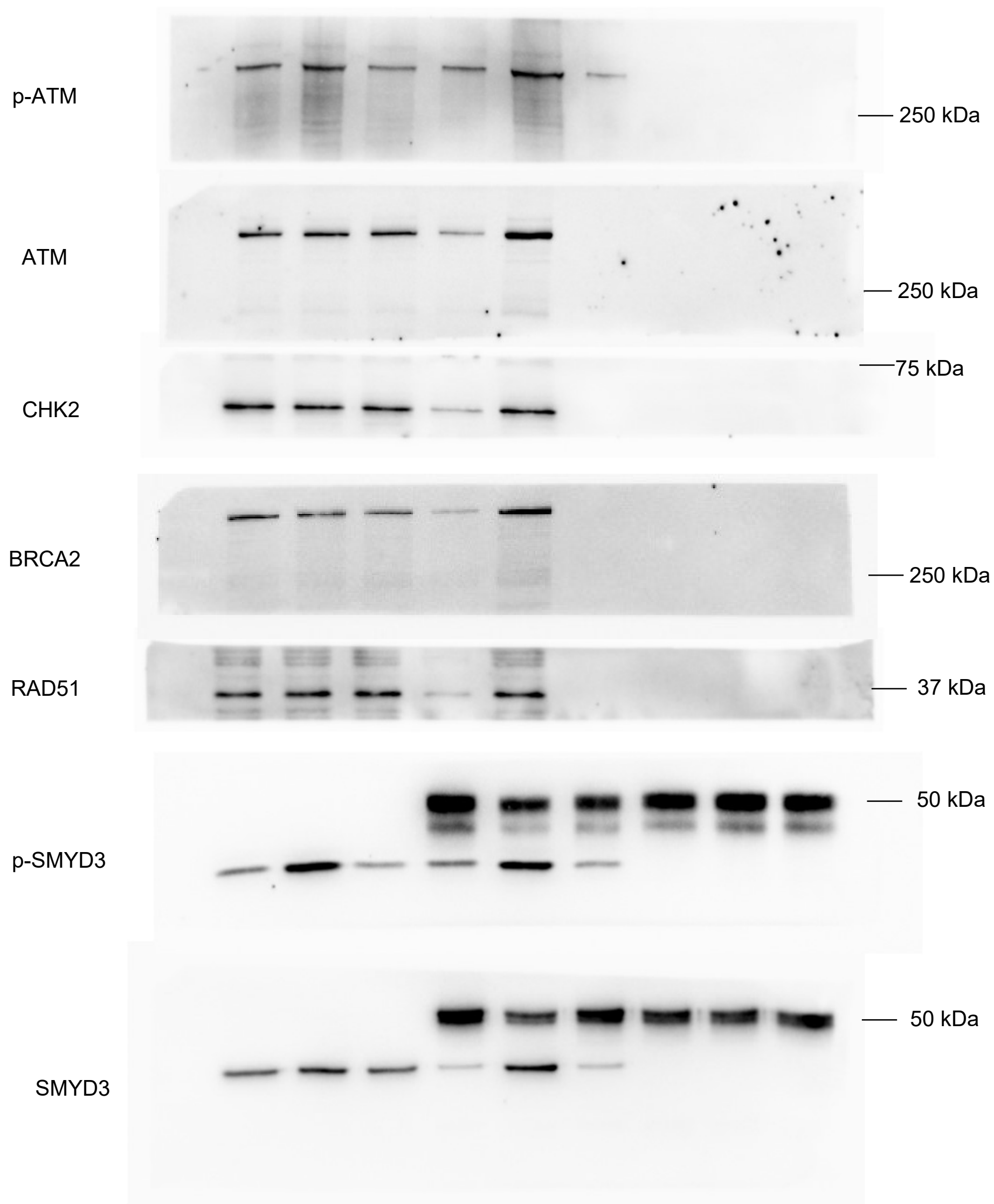
p-ATM
250 kDa
ATM
250 kDa
75 kDa
CHK2
BRCA2
250 kDa
RAD51
37 kDa
50 kDa
p-SMYD3
50 kDa
SMYD3

Figure 3g

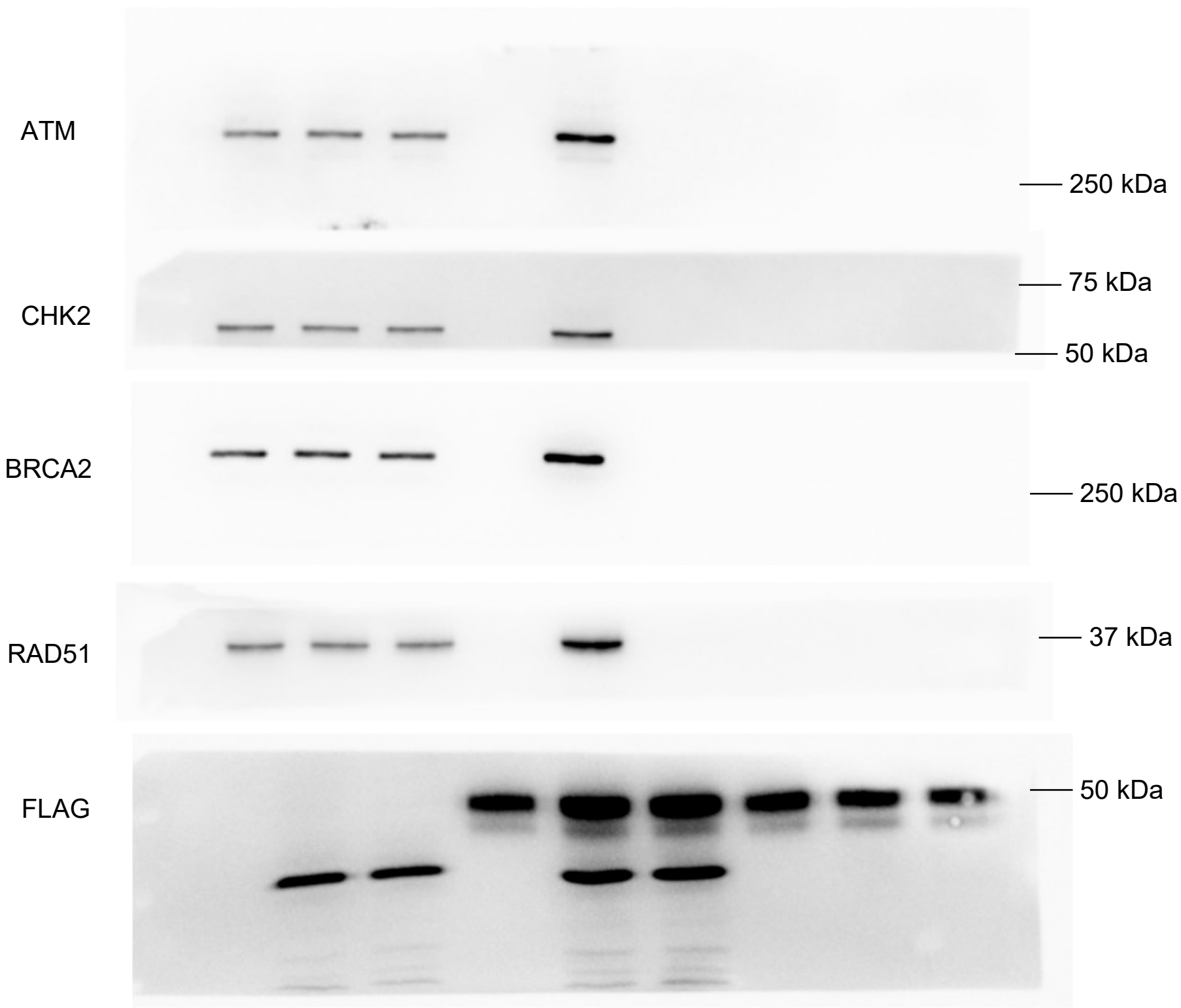
ATM
250 kDa
CHK2
75 kDa
50 kDa
BRCA2
250 kDa
RAD51
37 kDa
FLAG
50 kDa

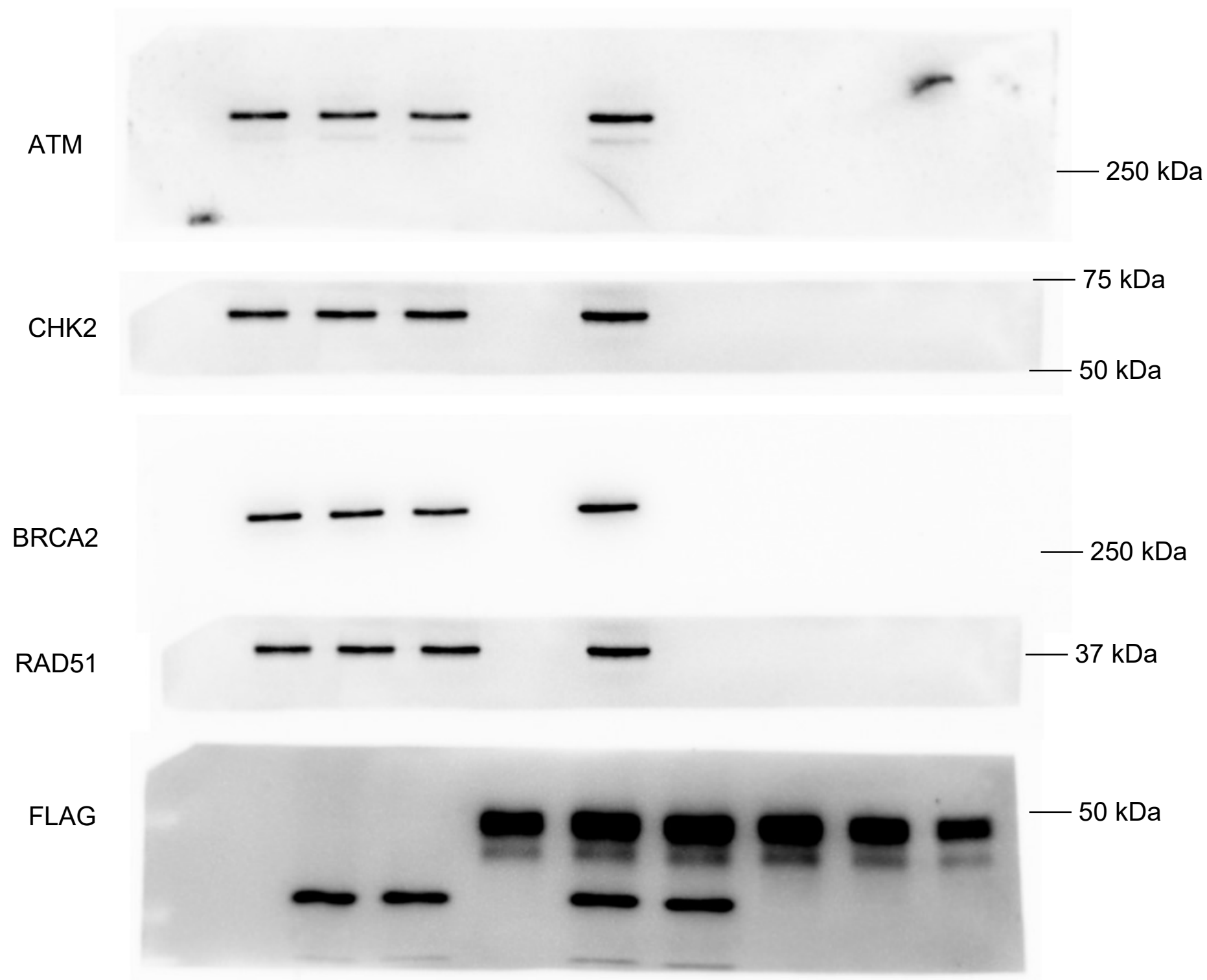
ATM
250 kDa
CHK2
75 kDa
50 kDa
BRCA2
250 kDa
RAD51
37 kDa
FLAG
50 kDa

Figure 4e

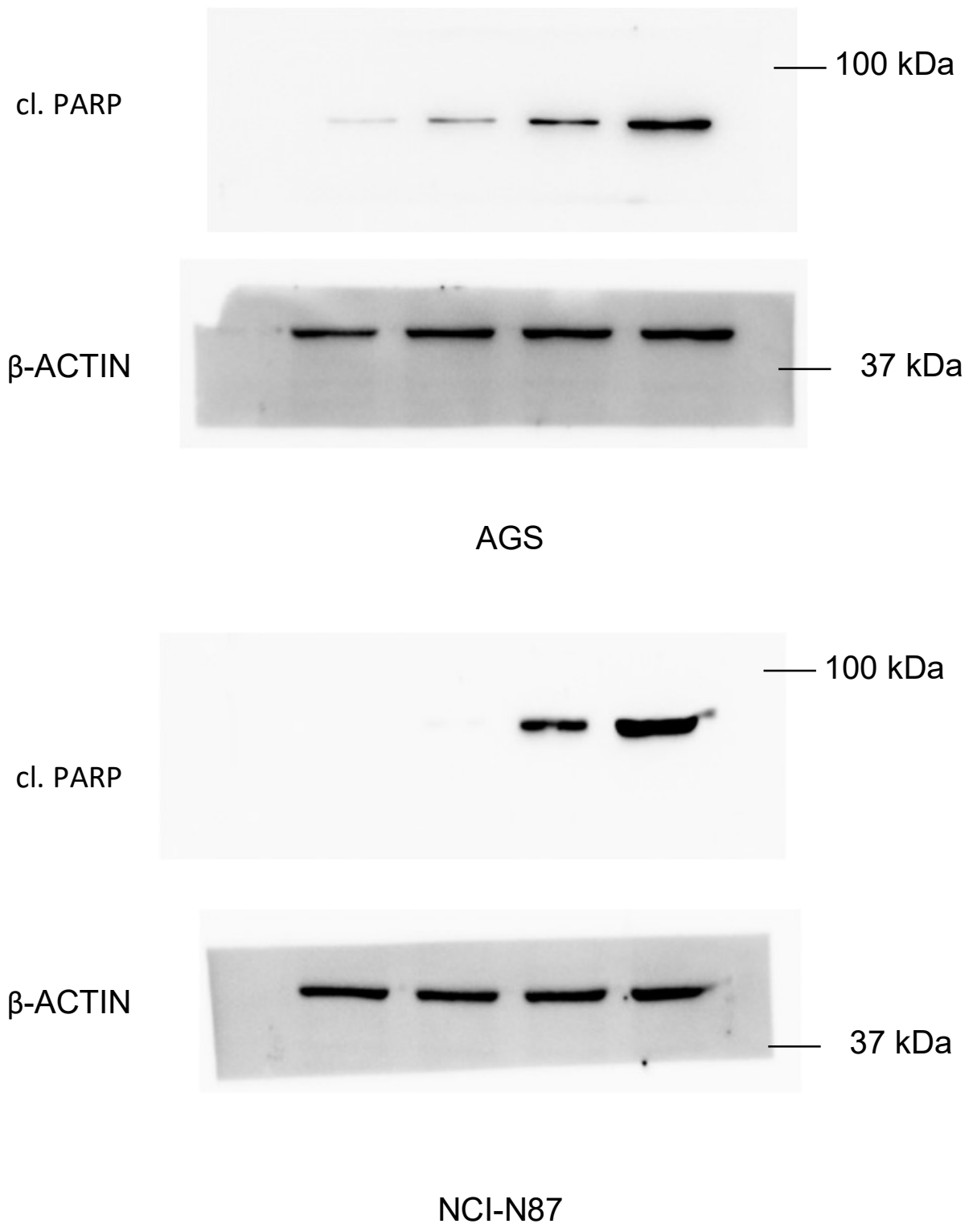

Figure 4f

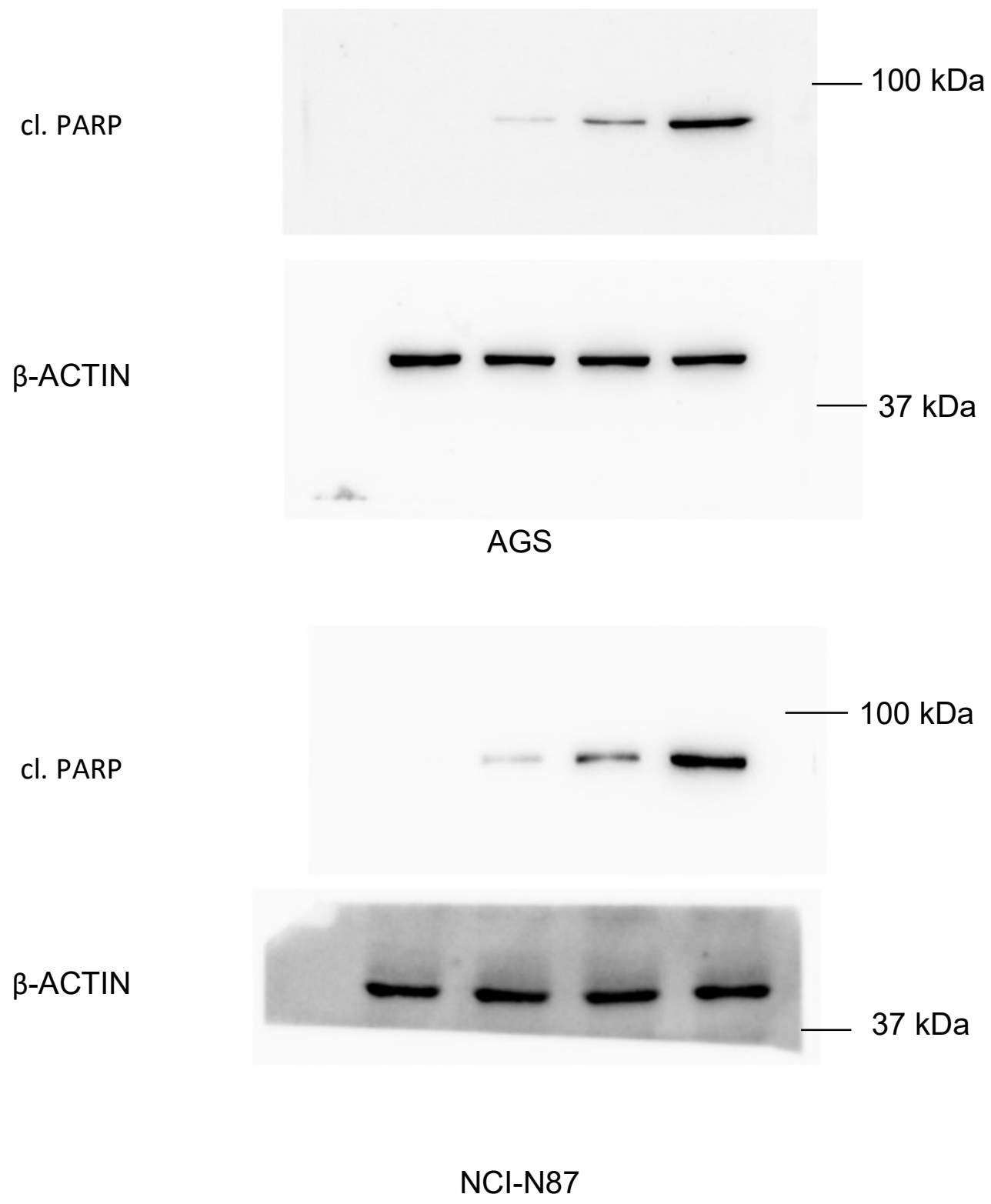

Figure 4k

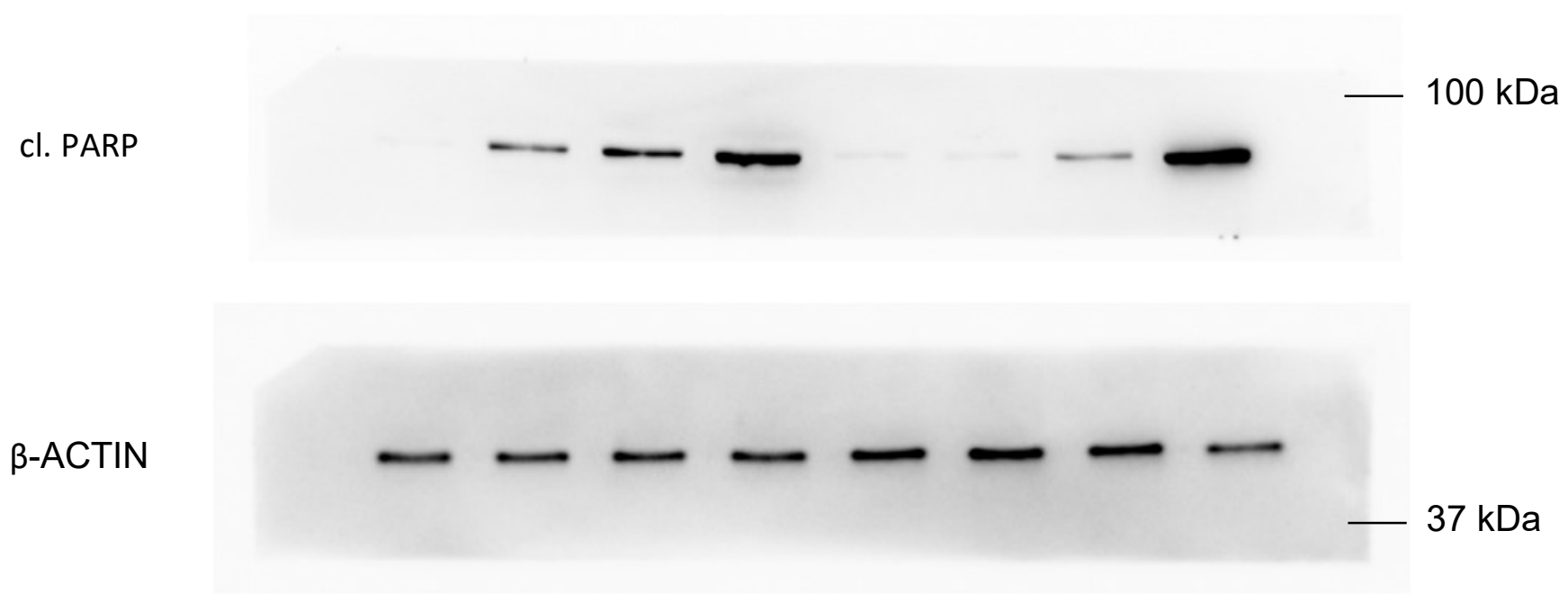

Figure 5d

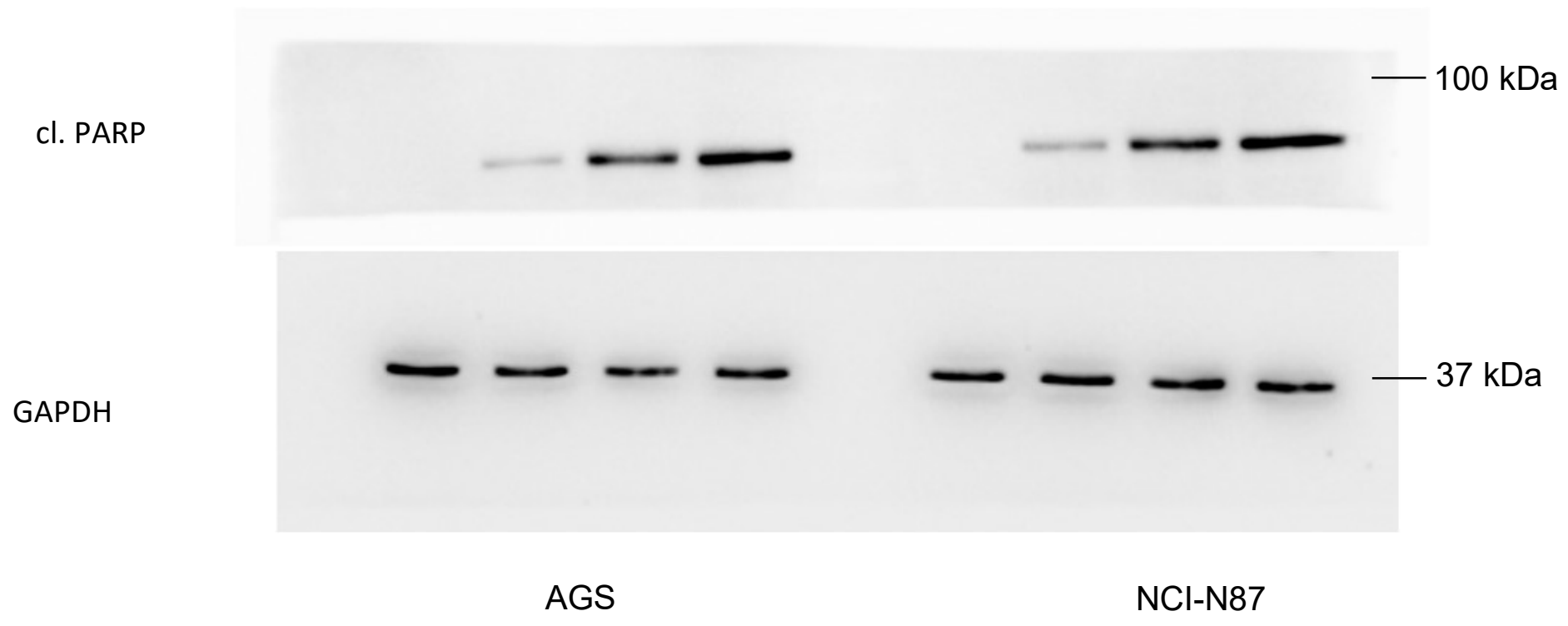

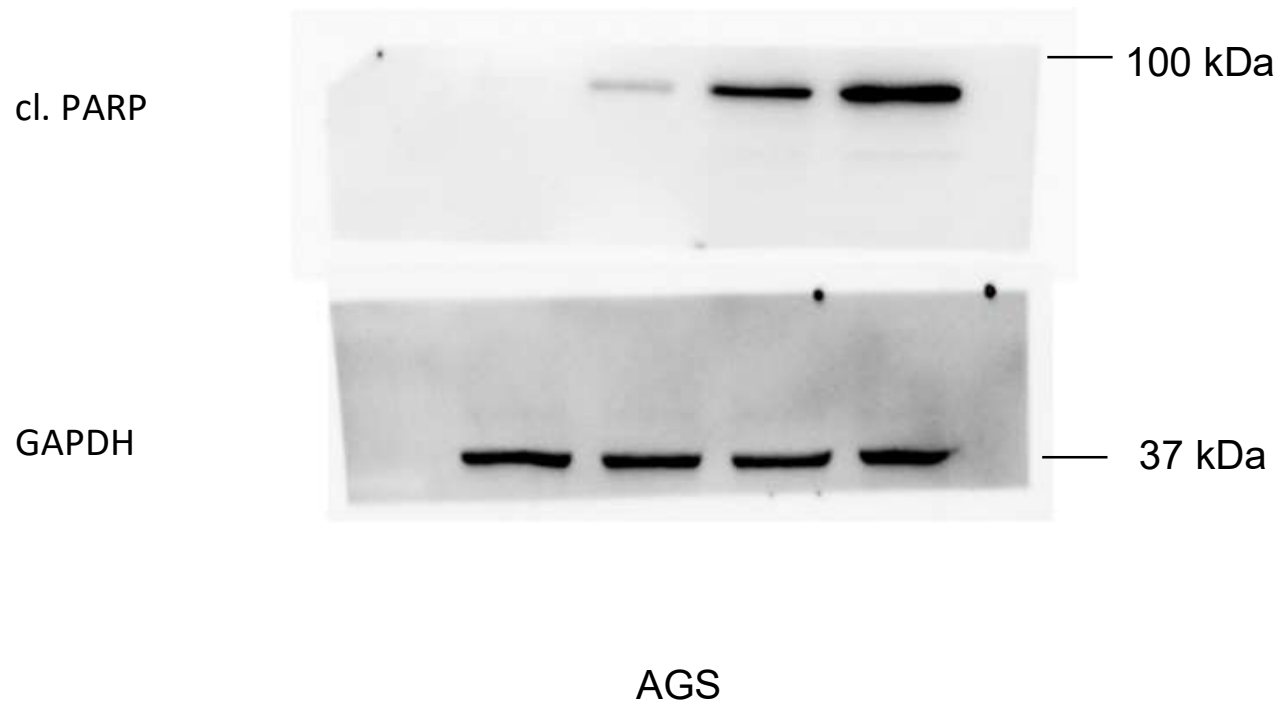

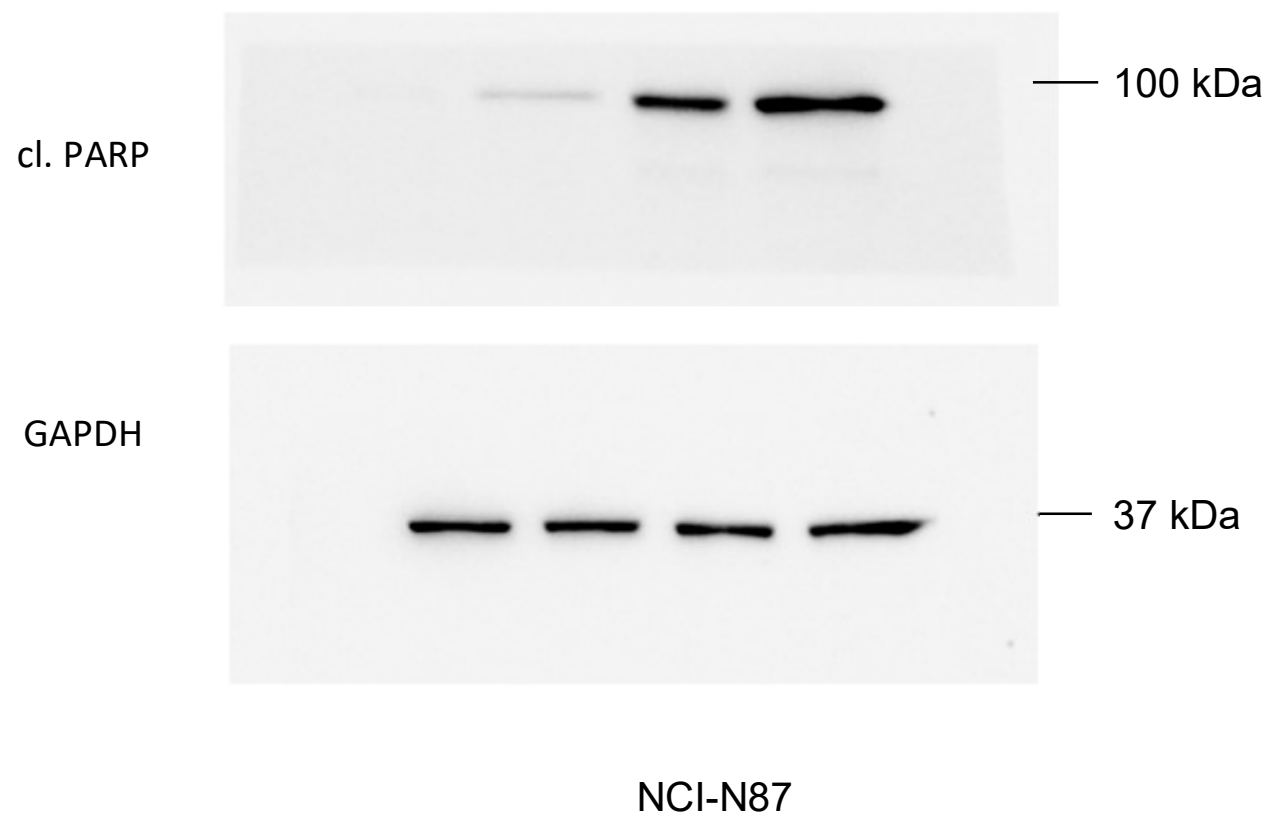

Figure 5g

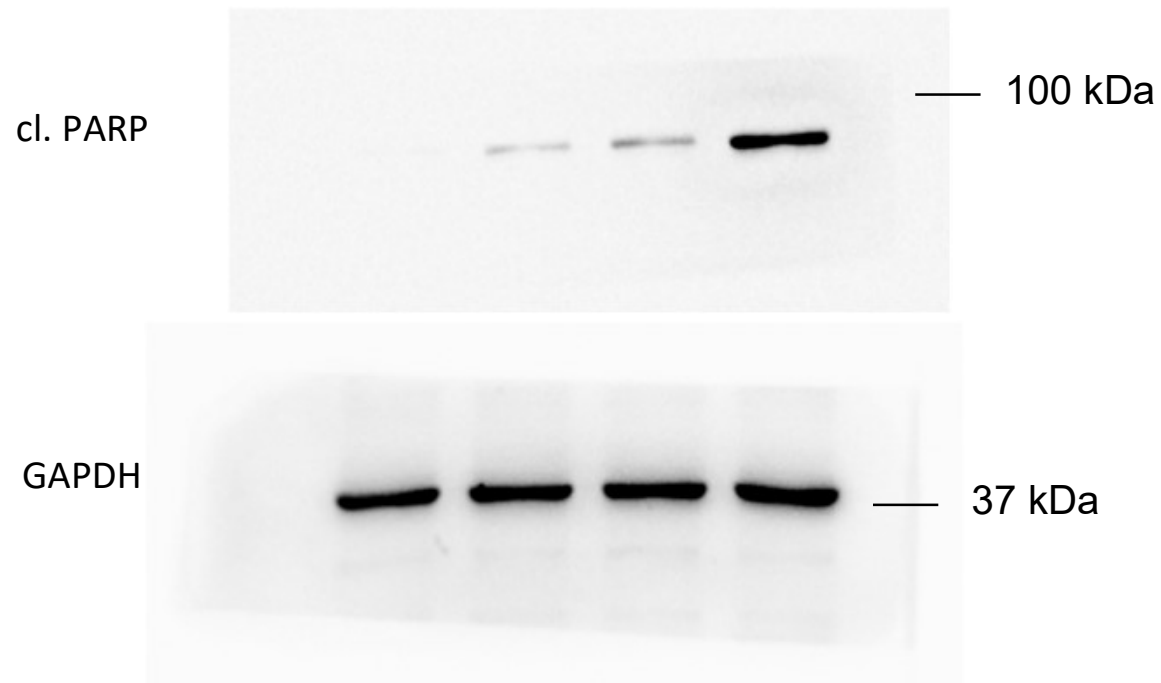

Figure 6f

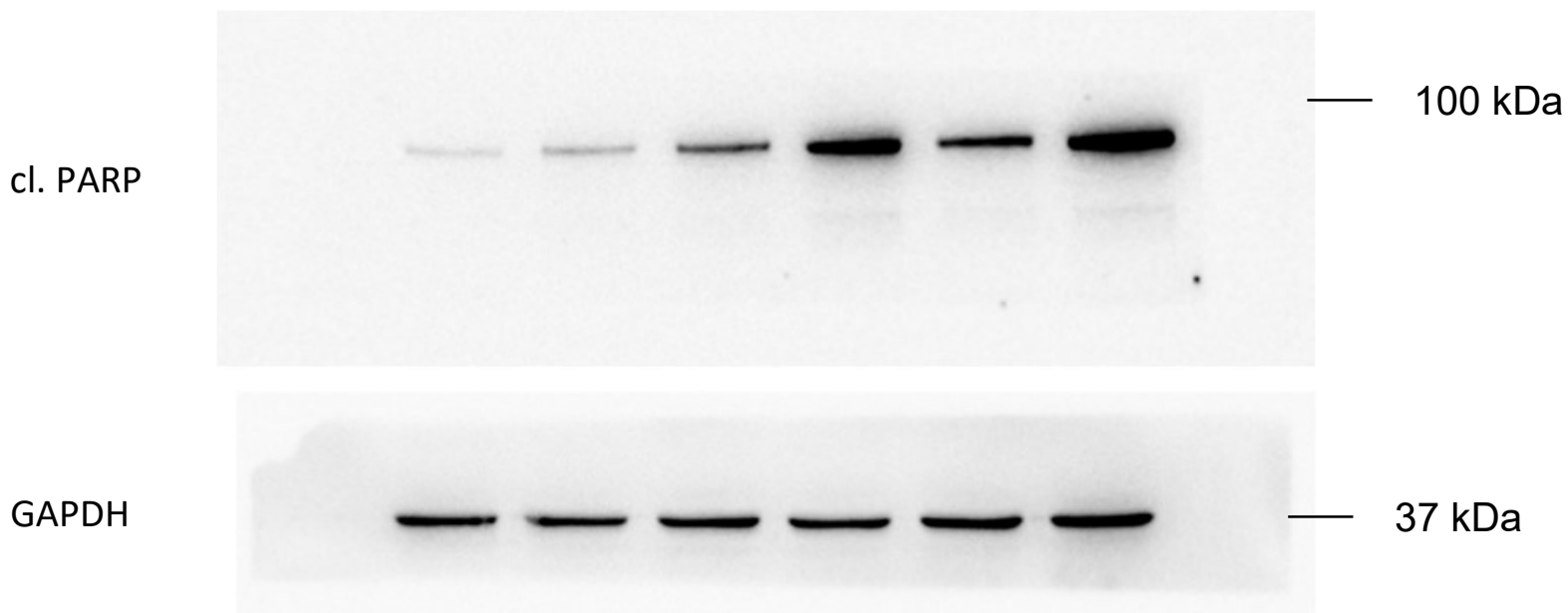

Supplementary Fig. 1c

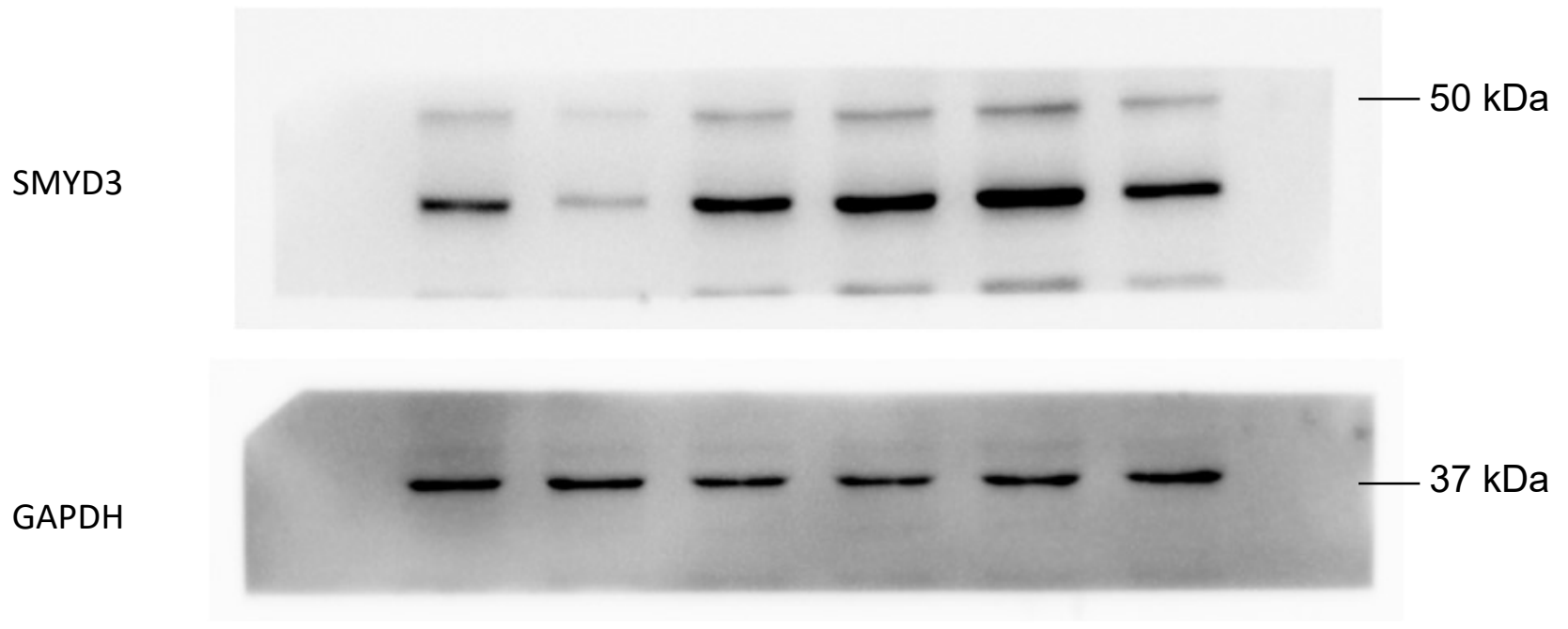

Supplementary Fig. 1e

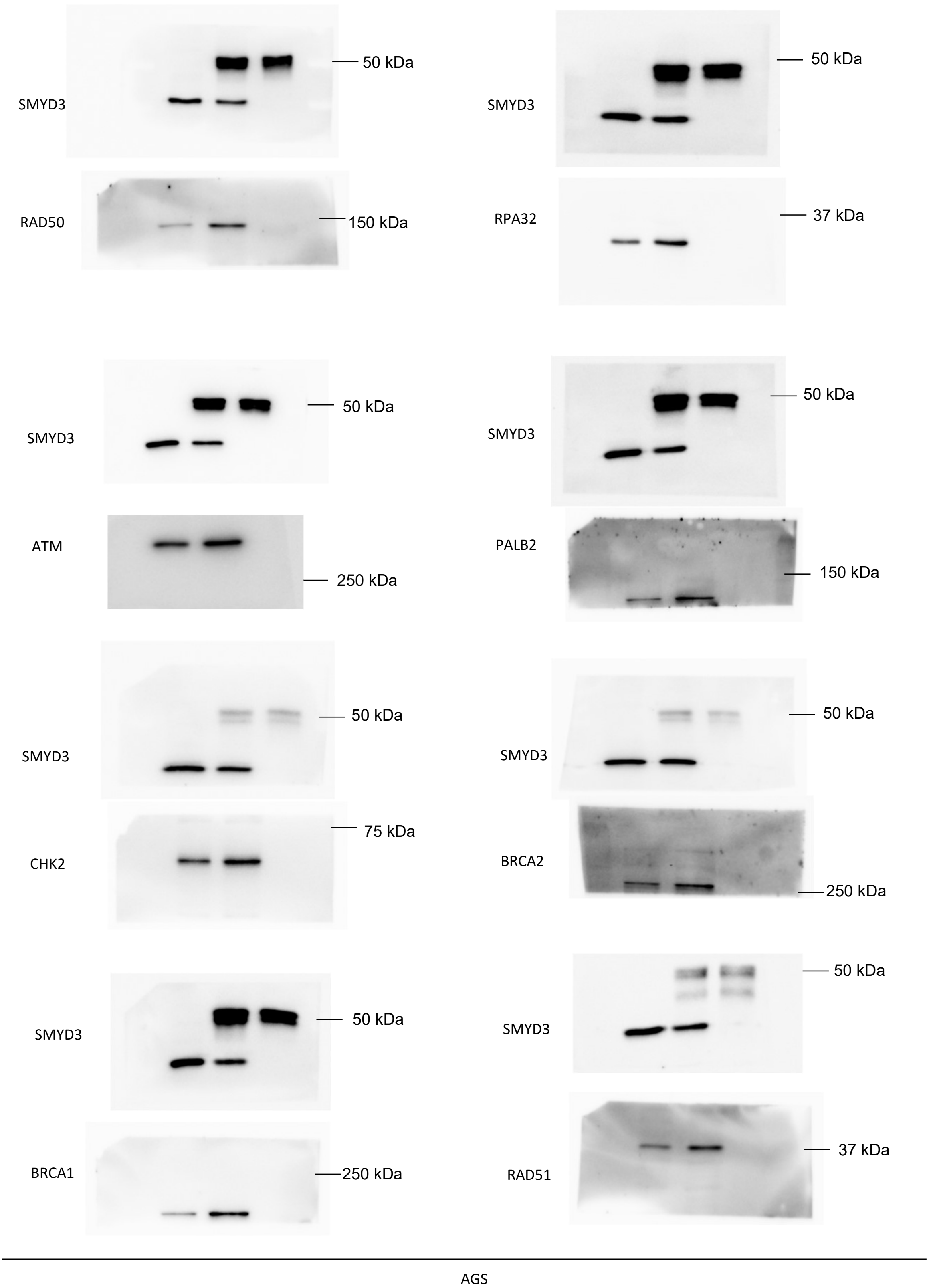

Supplementary Fig. 1e

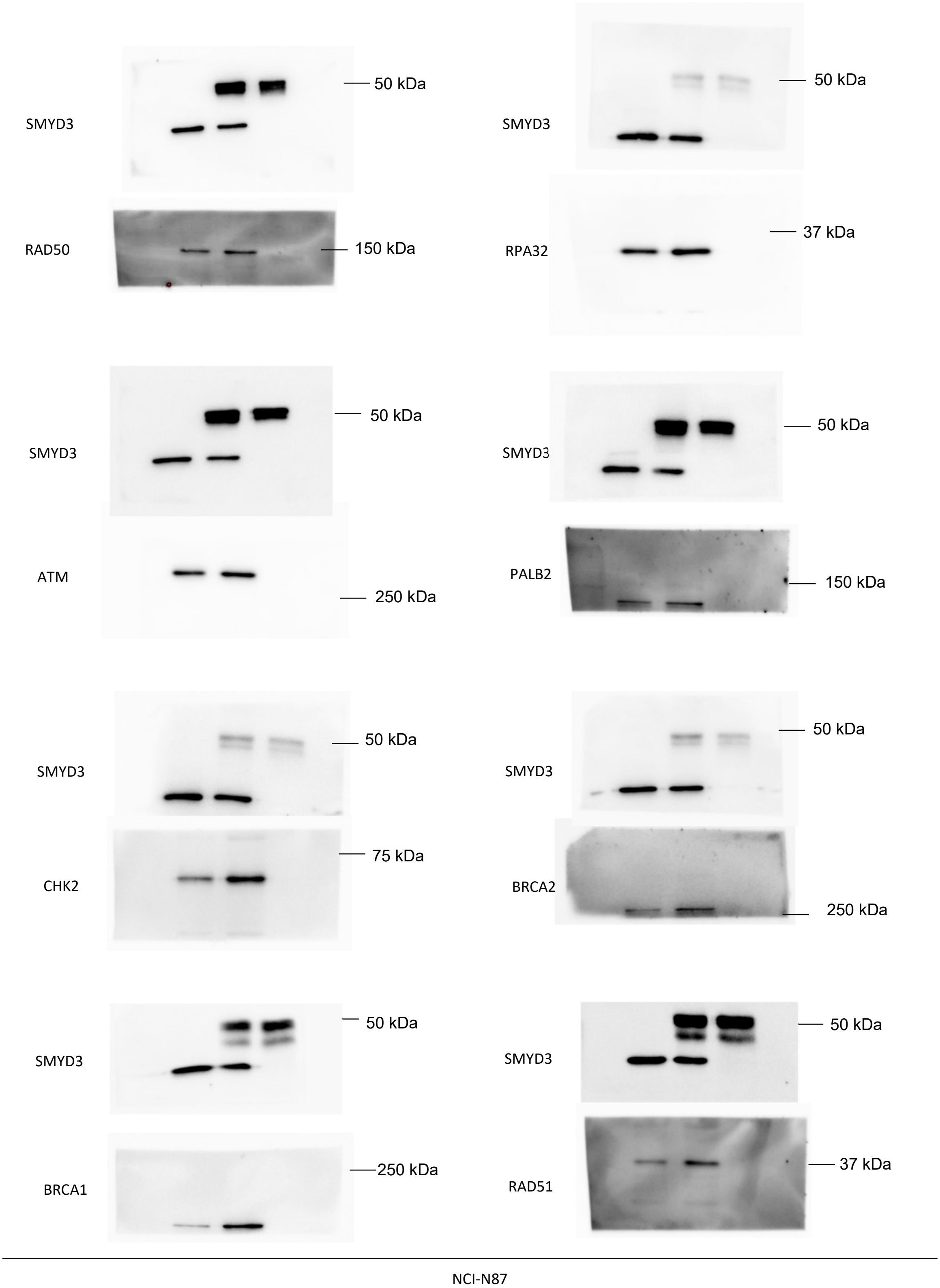

Supplementary Fig. 2f

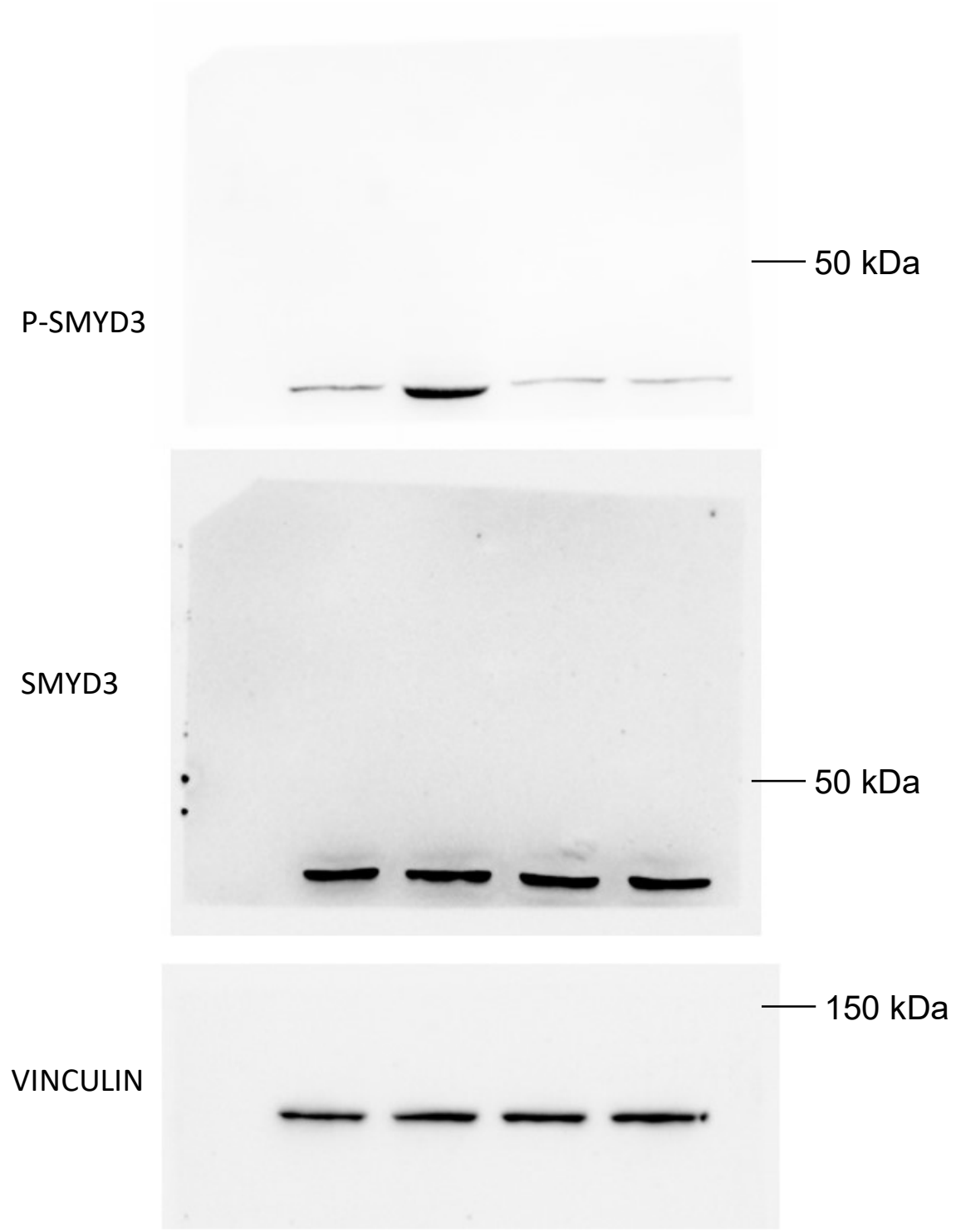

Supplementary Fig. 2h

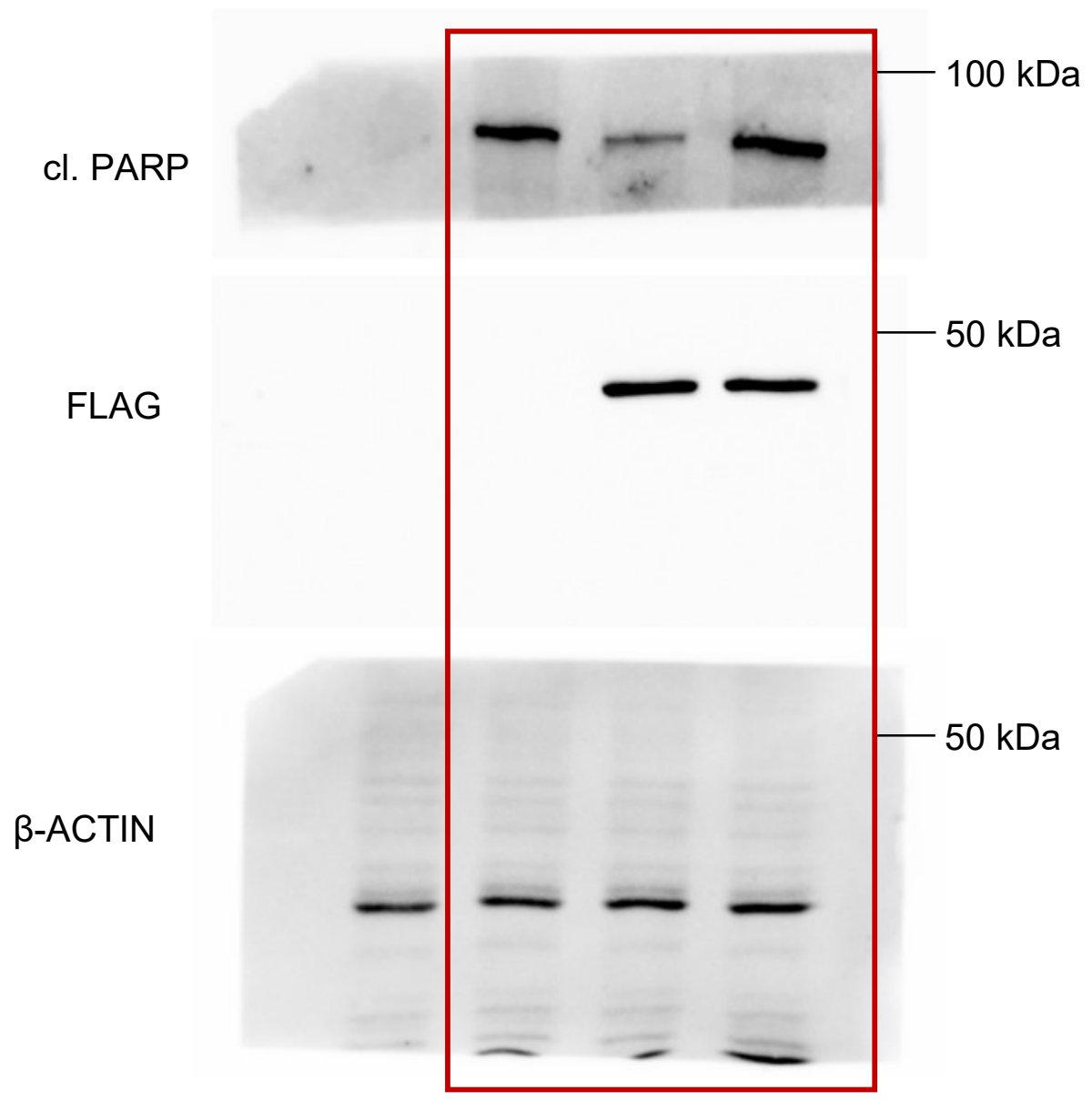

Supplementary Fig. 2j

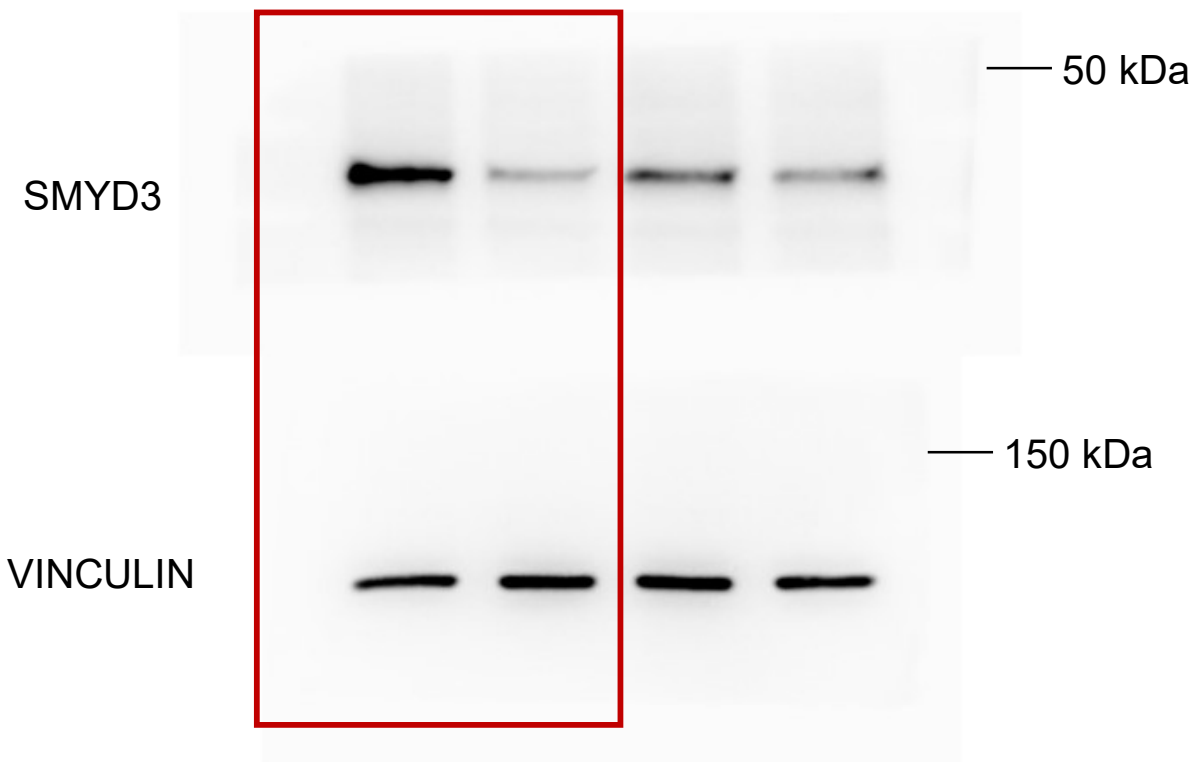

Supplementary Fig. 3a

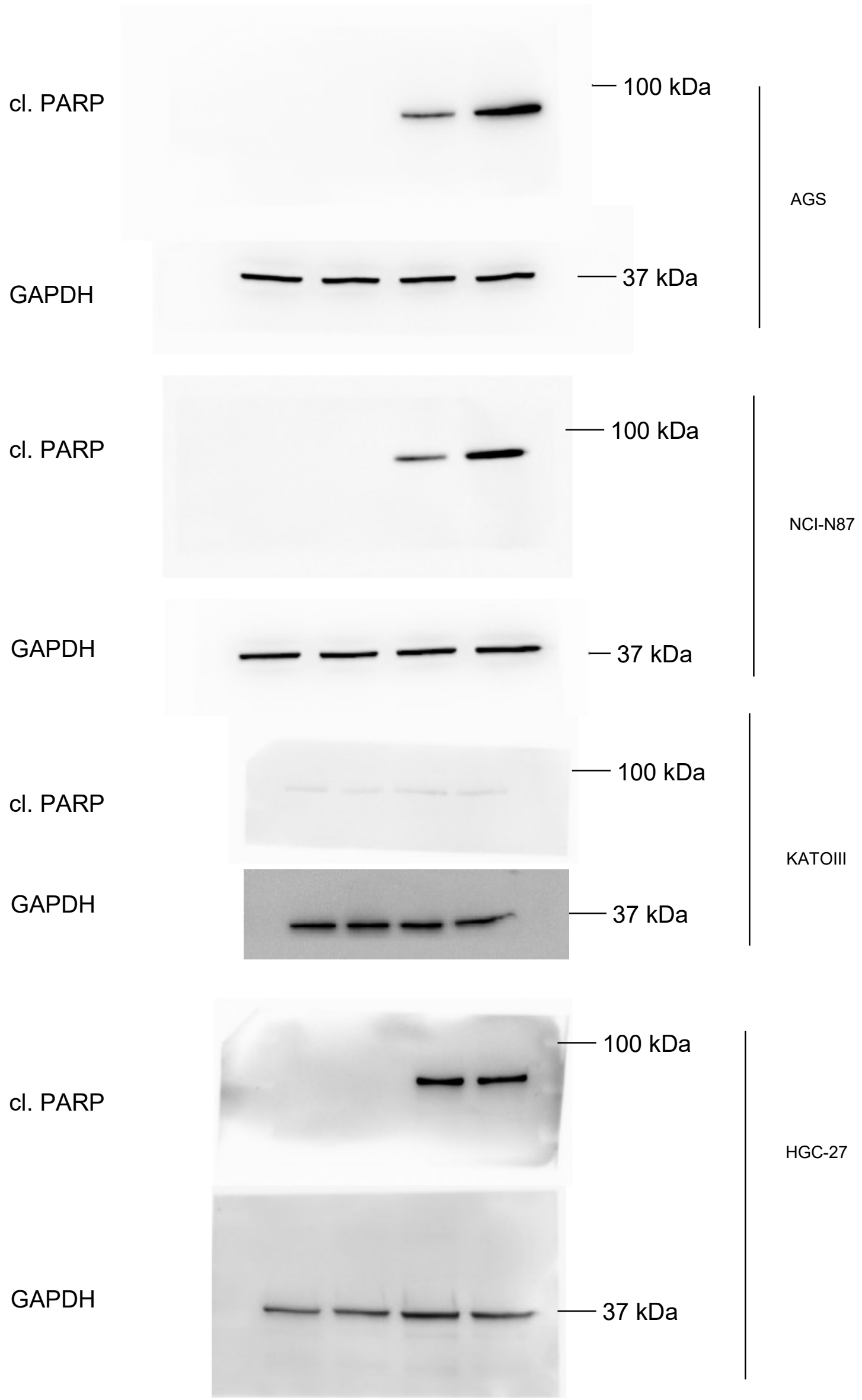

Supplementary Fig. 3j

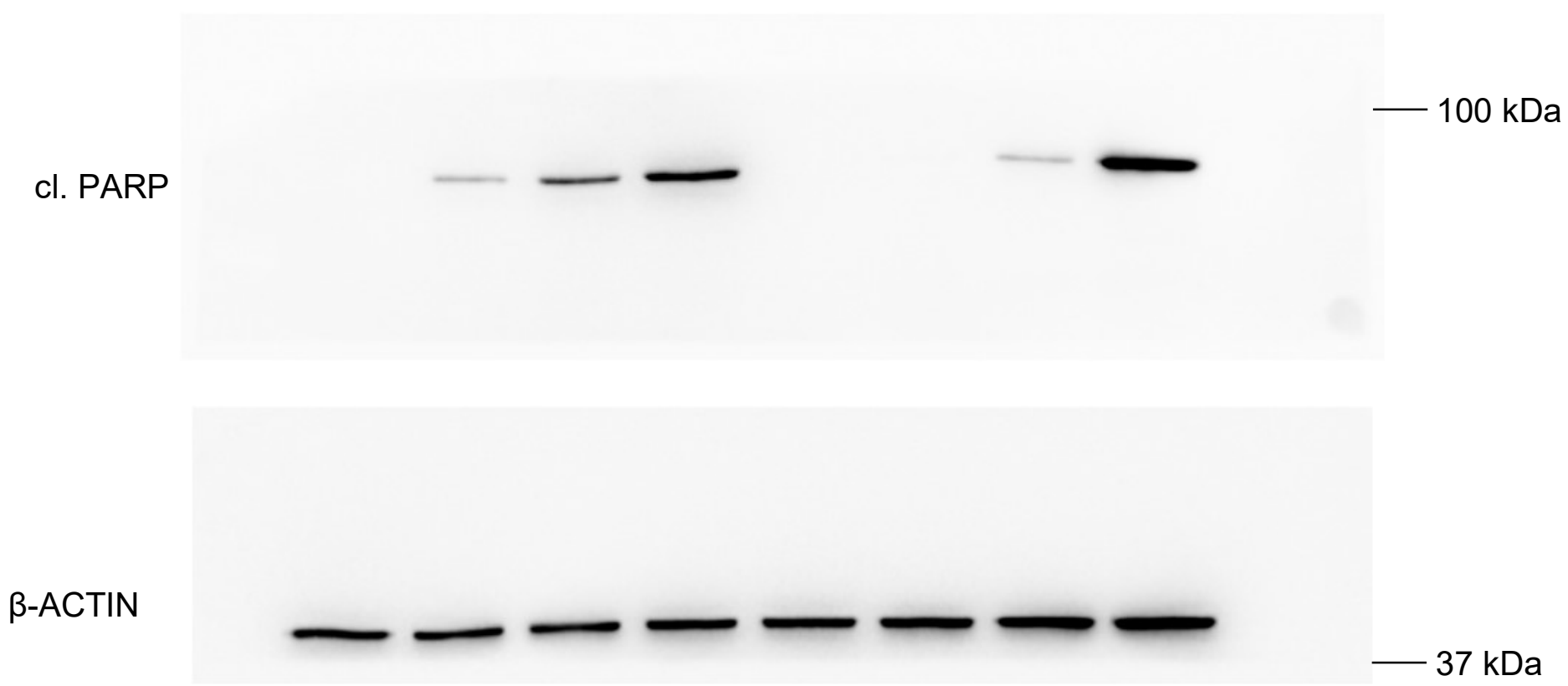

Supplementary Fig. 4a

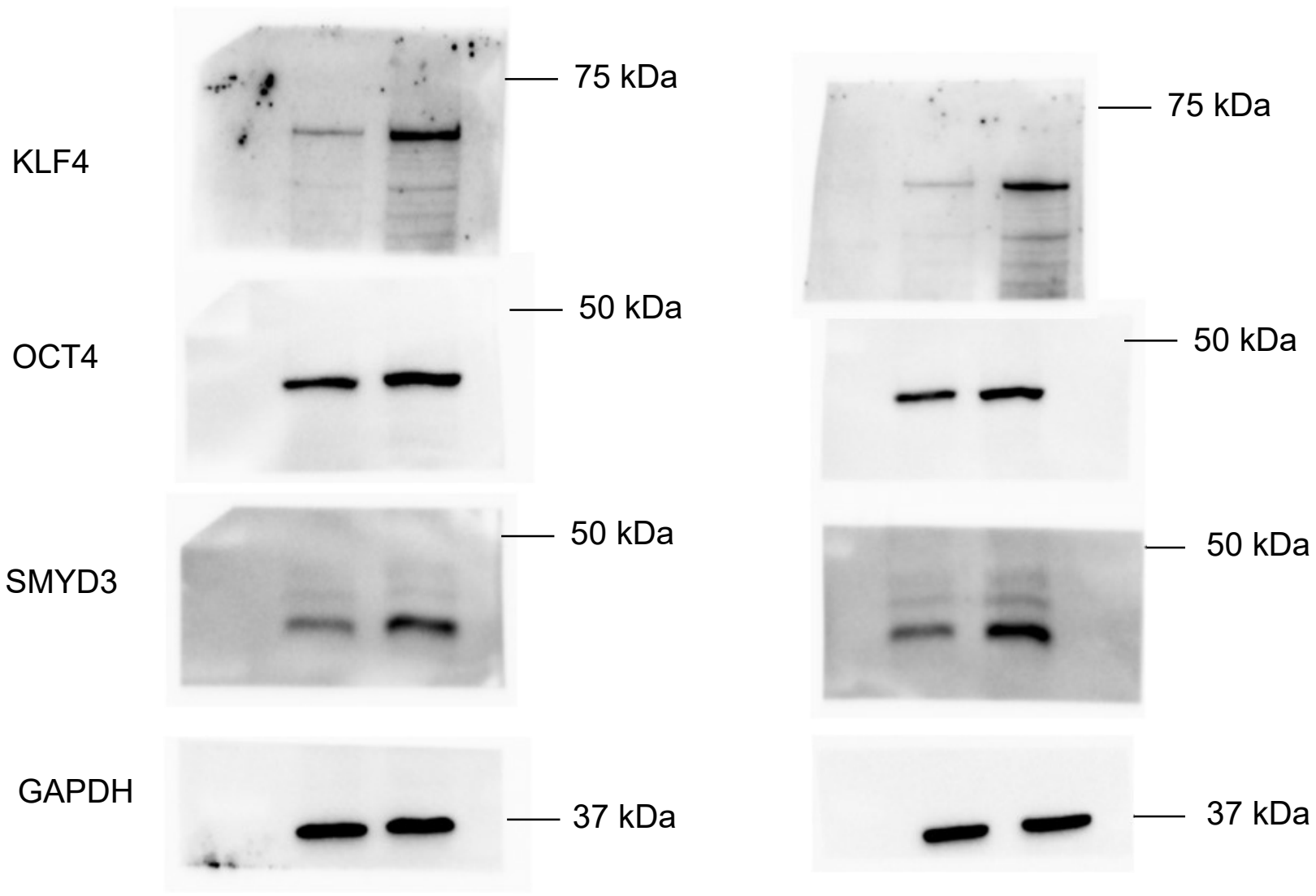

Supplement: Supplementary file 1 [file 13046_2026_3823_MOESM5_ESM.pdf]
